# VprBP/DCAF1 regulates p53 function and stability through site-specific phosphorylation

**DOI:** 10.1038/s41388-023-02685-8

**Published:** 2023-04-11

**Authors:** Nikhil Baban Ghate, Sungmin Kim, Roasa Mehmood, Yonghwan Shin, Kyunghwan Kim, Woojin An

**Affiliations:** 1 Department of Biochemistry and Molecular Medicine, Norris Comprehensive Cancer Center, University of Southern California, Los Angeles, CA 90033 USA; 2 Department of Biological Sciences and Biotechnology, Chungbuk National University, Cheongju, Chungbuk Republic of Korea

**Keywords:** Oncogenes, Cancer prevention

## Abstract

VprBP (also known as DCAF1) is a recently identified kinase that is overexpressed in cancer cells and serves as a major determinant for epigenetic gene silencing and tumorigenesis. The role of VprBP in driving target gene inactivation has been largely attributed to its ability to mediate histone H2A phosphorylation. However, whether VprBP also phosphorylates non-histone proteins and whether these phosphorylation events drive oncogenic signaling pathways have not been explored. Here we report that serine 367 phosphorylation (S367p) of p53 by VprBP is a key player in attenuating p53 transcriptional and growth suppressive activities. VprBP catalyzes p53S367p through a direct interaction with the C-terminal domain of p53. Mechanistically, VprBP-mediated S367p inhibits p53 function in the wake of promoting p53 proteasomal degradation, because blocking p53S367p increases p53 protein levels, thereby enhancing p53 transactivation. Furthermore, abrogation of VprBP-p53 interaction by p53 acetylation is critical for preventing p53S367p and potentiating p53 function in response to DNA damage. Together, our findings establish VprBP-mediated S367p as a negative regulator of p53 function and identify a previously uncharacterized mechanism by which S367p modulates p53 stability.

## Introduction

p53 is an important tumor suppressor controlling a wide range of DNA damage response processes, and its functional inactivation is the most frequent alteration in human cancers [1, 2]. Although some p53 effects may involve nontranscriptional mechanisms, major roles played by p53 are mediated through its action as a transcription factor that regulates the expression of downstream target genes such as p21, BTG2, and PUMA [3–8]. Structurally and functionally, p53 can be divided into four major domains: the N-terminal transactivation domain (residues 1–80), the central DNA binding domain (residues 100–290), the C-terminal tetramerization domain (residues 323–355), and the C-terminal regulatory domain (residues 364–393) [9–11]. All these domains are uniquely dedicated to a rapid and precise action of p53 on its target genes. In unstressed cells, p53 levels are very low owing to rapid degradation via ubiquitin-proteasome pathways in the cytoplasm. In the event of DNA damage, p53 becomes stabilized and activated to control the expression of target genes in the nucleus [12]. The exact mechanism of the enhancement of p53 function upon DNA damage is not yet fully understood, but accumulating evidence indicates that p53 activity is influenced by posttranslational modifications [13–17]. Among all known modifications, phosphorylation and acetylation are so far the most intensively studied modifications of p53 [15, 18–24]. They are dynamically regulated after DNA damage and widely accepted to be crucial for controlling the stability and sequence specific DNA binding capacity of p53, thereby affecting p53-dependent transcriptional program. Moreover, while initially thought to act independently, accumulating evidence suggest that p53 phosphorylation and acetylation can work together in a mutually exclusive or cooperative manner [14, 25–31]. Thus, it seems of importance to unravel molecular mechanisms and signaling pathways that govern the actions of these modifications in establishing thresholds for p53 activation and target gene expression in response to DNA damage.

The HIV-1 Vpr Binding Protein (VprBP), also known as DDB1 and CUL4 Associated Factor 1, is a nuclear-localized protein and was originally identified owing to its interaction with HIV-1 Vpr in the context of the HIV-1 life cycle [32, 33]. Since its discovery, VprBP has been mainly characterized as the substrate recognition component of E3 ubiquitin ligase complexes and implicated in regulating cell cycle progression and DNA replication [34, 35]. Of special relevance to the current study, our recent investigation unexpectedly revealed that VprBP participates in establishing a transcriptionally inactive chromatin state and impairs p53-dependent transactivation of target genes [36]. Additional support for the repressive action of VprBP came from cellular analyses showing that silencing VprBP expression leads to the upregulation of p53 downstream target genes. Our demonstration of physical interaction between VprBP and p53 in cellular environments suggested that VprBP may directly prevent p53 from functioning properly at target genes [36]. However, this interaction model does not explain the molecular mechanism by which VprBP mediates transrepression of other genes apart from p53 responsive genes. Our continued efforts toward addressing this key question led to the finding that VprBP possesses an intrinsic kinase activity and catalyzes histone H2A T120 phosphorylation (H2AT120p) to repress transcription in cancer cells [37]. A role for VprBP-mediated H2AT120p in gene silencing is directly supported by the observation that a point mutation of T120 in H2A eliminates the ability of VprBP to establish an inactive transcription state. Also, kinase-dead mutations almost completely abolished the transrepression potential of VprBP in cancer cells, implying H2AT120p-dependent mechanism for VprBP function in negatively regulating transcription and inducing oncogenic transformation. Given the significance of VprBP-mediated H2AT120p in gene silencing, we also developed a small molecule inhibitor, named B32B3, capable of selectively targeting VprBP and blocking its kinase activity in cancer cells [37, 38]. While all these data emphasize the oncogenic property of VprBP-mediated H2AT120p, it remains elusive whether phosphorylation of non-histone proteins also plays a critical role in VprBP-driven oncogenic events. This question is important since VprBP might fulfill its function by physically targeting gene regulatory factors and influencing their activities at particular genomic loci.

Here, we demonstrate an intrinsic ability of VprBP to phosphorylate p53 and suppress p53 transcriptional activity, thereby diminishing the expression of target genes. Specifically, VprBP directly interacts with p53 and catalyzes S367p to promote p53 proteasomal degradation. Moreover, we show that p53 acetylation in response to DNA damage inhibits VprBP kinase activity toward p53S367 by blocking VprBP binding to p53 and increases p53 stability to potentiate target gene expression. Our studies uncover a previously unrecognized role for VprBP in modulating p53 protein stability and may provide the basis for designing therapeutic approaches that restore p53 function in cancer cells.

## Results

### VprBP associates with p53 and catalyzes S367p in vitro

**Fig. 1:**
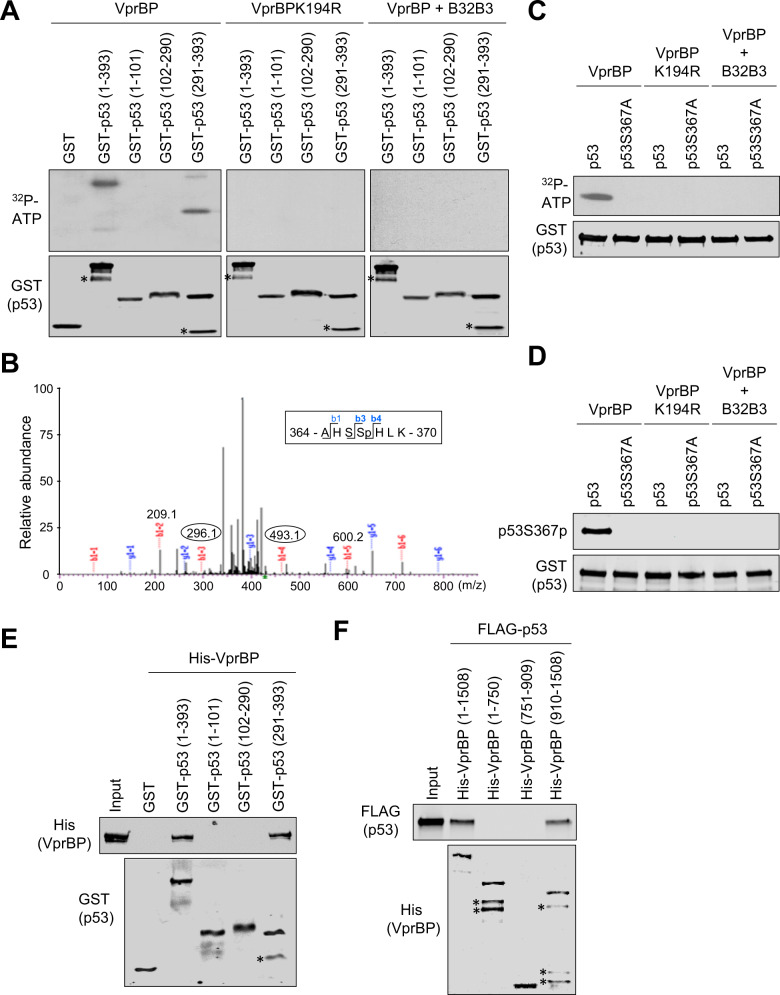
Direct phosphorylation of p53 at S367 by VprBP. **A** The indicated recombinant GST-tagged p53 proteins were incubated with VprBP or VprBPK194R in the presence or absence of VprBP kinase inhibitor B32B3 and [γ-^32^P] ATP for 30 min. The reactions were then resolved on 5–20% SDS-PAGE and analyzed by autoradiography (upper panel) and Western blot (lower panel). **B** p53 251-374 was phosphorylated by VprBP, digested with trypsin, and analyzed by liquid chromatography-tandem mass spectrometry (LC-MS/MS) to identify phosphorylation sites. **C** p53 wild-type or S367A mutant proteins were incubated with VprBP or VprBPK194R in presence or absence of VprBP kinase inhibitor B32B3 and [γ-^32^P] ATP for 30 min. The modified p53 proteins were analyzed by autoradiography (upper panel) and Western blot (lower panel). **D** Reactions from (**C**) were analyzed by Western blot using the antibody raised against p53S367p. (See also Supplementary Fig. S1). **E** GST alone or the indicated GST‐p53 fusions were immobilized on glutathione‐Sepharose beads and incubated with His‐VprBP. After washing, bound VprBP proteins were fractionated by SDS/PAGE and examined by Western blotting with anti‐His antibody. **F** Indicated His‐VprBP fusions were immobilized on Nickel beads and incubated with FLAG‐tagged p53. After washing, bound p53 proteins were detected by Western blotting with anti‐FLAG antibody.

We previously reported that VprBP has an intrinsic protein kinase activity capable of phosphorylating histone H2A in cancer cells [37]. This result and our demonstration of VprBP function in suppressing p53-dependent transcription raised the possibility that VprBP might target p53 as a direct substrate for its function. In addressing this question, we first conducted in vitro kinase assays by incubating bacterially expressed recombinant p53 proteins with VprBP and [γ-32P]-ATP. Autoradiographic analysis of the reactions demonstrated that VprBP can phosphorylate p53 (Fig. 1A, VprBP). Because p53 contains N-terminal transactivation (residues 1-80), central DNA binding (residues 102-290), and C-terminal multifunctional (residues 291-393) domains, kinase assays were repeated with these well-characterized domains. In these assays, VprBP generated a clear radiolabeling of p53 C-terminal multifunctional domain, whereas it failed to do so on p53 N-terminal transactivation and central DNA binding domains (VprBP). When kinase assays were performed with VprBPK194R kinase-dead mutant (VprBPK194R) or in the presence of VprBP inhibitor B32B3 (VprBP + B32B3), we failed to see p53 phosphorylation in all reactions, confirming the specificity and integrity of our kinase reactions.

As an approach toward mapping phosphorylation sites, we next carried out mass spectrometric sequencing on the p53 293-393 fragment after the phosphorylation reaction. Our analysis identified only a singly phosphorylated peptide that could have arisen from phosphorylation of serine 367 (S367) (Fig. 1B). Consistent with the mass spectrometric data, alanine substitution of S367 (S367A) almost completely abrogated VprBP-mediated p53 phosphorylation (Fig. 1C). To confirm and analyze in more detail p53S367 phosphorylation (p53S367p), we raised a rabbit polyclonal antibody that reacts with p53S367p. The specificity of the purified antibody was verified by dot blot and peptide competition assays using p53 unmodified and S367p peptides (Supplementary Fig. S1). In Western blot analysis of the kinase reactions, this p53S367p antibody reacted strongly with p53 wild-type, but not with p53S367A (Fig. 1D, VprBP). Moreover, inclusion of VprBP inhibitor B32B3 in kinase reactions efficiently blocked p53S367p, indicating that VprBP is responsible for the observed p53S367p (Fig. 1D, VprBP + B32B3).

Although our in vitro modification assays confirmed the ability of VprBP to catalyze p53S367p, it is not clear whether VprBP kinase activity toward p53 requires its physical interaction with p53. To check this possibility, we conducted in vitro pull-down assays using His-tagged VprBP and glutathione S-transferase (GST)-fused p53. As shown in Fig. 1E, GST-p53 efficiently interacted with His-VprBP, whereas GST alone did not. When binding experiments were repeated with the N-terminal, central, and C-terminal domains of p53, VprBP interacted with p53 C-terminal multifunctional domain (residues 291-393), while no apparent interaction was observed with p53 N-terminal transactivation (residues 1-101) and central DNA binding (residues 102-290) domains (Fig. 1E). In mapping p53-interacting region of VprBP, the direct binding of VprBP C-terminal domain (residues 910-1580) was readily detectable, but VprBP N-terminal (residues 1-750) and central (residues 751-909) domains showed no interaction with p53 (Fig. 1F), strongly supporting that the C-terminal domain plays a major role in VprBP binding to p53.

### VprBP is the kinase responsible for cellular p53S367p and inactivation

**Fig. 2:**
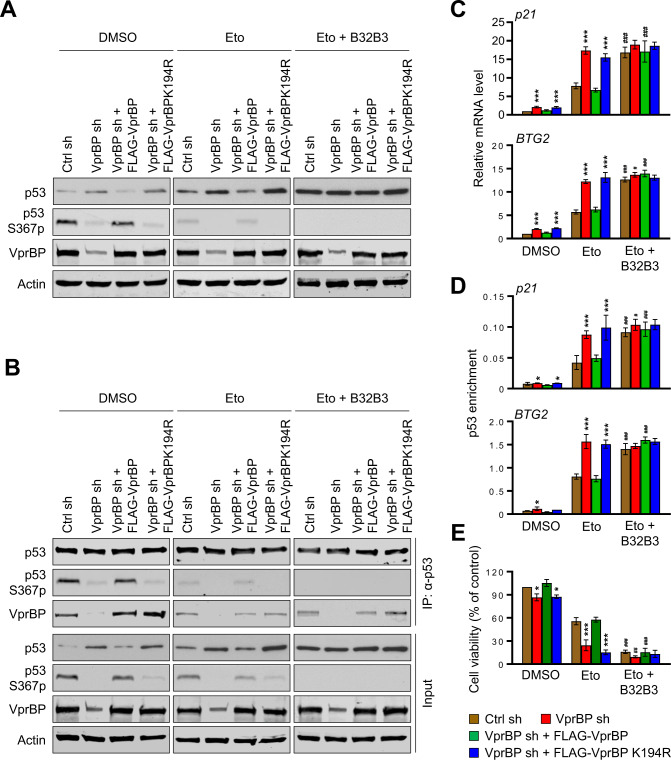
p53 protein level and transcriptional activities altered after S367p. **A** Control, VprBP-depleted, and VprBP/VprBPK194R-rescued U2OS cells were treated with etoposide (40 µM) in the presence or absence of B32B3 (0.5 µM) for 24 h. Whole cell lysates were prepared and subjected to Western blotting using p53, p53S367p and VprBP antibodies. **B** Whole cell lysates from (**A**) were subjected to immunoprecipitation using p53 DO-1 antibody. The binding of VprBP to p53 was analyzed by Western blotting. **C** Total RNA was isolated from the cells treated as in (**A**), and subjected to RT-qPCR analysis with p21 and BTG2 specific primers listed in Supplementary Table S1. Data represent the means ± SD of three independent experiments. *P* values were calculated using two-way ANOVA with post-hoc Tukey’s test for multiple comparisons. ***P < 0.001 versus Ctrl sh; ^#^*P* < 0.05 and ^###^*P* < 0.001 versus Eto. (See also Supplementary Fig. S4A). **D** ChIP assays were performed in the U2OS cells treated as in (**A**) using p53 DO-1 antibody. Precipitated DNA was amplified with primers listed in Supplementary Table S2. Data are represented as mean ± SD of three independent experiments. *P* values were calculated using two-way ANOVA with post-hoc Tukey’s test for multiple comparisons. *P < 0.05 and ***P < 0.001 versus Ctrl sh; ^#^*P* < 0.05 and ^###^*P* < 0.001 versus Eto. (See also Supplementary Fig. S4B). **E** U2OS cells were treated as in (**A**) and their growth was assessed after 72 h culture using the cell proliferation reagent WST-1. Data are represented as mean ± SD of three independent experiments. *P* values were calculated using two-way ANOVA with post-hoc Tukey’s test for multiple comparisons. *P < 0.05 and ***P < 0.001 versus Ctrl sh; ^##^*P* < 0.01 and ^###^*P* < 0.001 versus Eto.

Based on the capacity of VprBP to generate p53S367p in vitro, we next explored whether VprBP can also catalyze cellular p53S367p and, if so, S367p has any significance in regulating p53 function in response to DNA damage. As confirmed by Western blotting, VprBP is readily detectable in U2OS human osteosarcoma cells, and shows little changes in its levels following treatment with etoposide and doxorubicin which are topoisomerase II-targeted, DNA cleavage-inducing agents (Fig. 2A, Supplementary Fig. S2A, VprBP, Eto, and Dox). Contrarily, a reproducible increase in p53 protein levels was observed in response to the etoposide/doxorubicin-induced DNA damage (p53, Eto, and Dox). In Western blot analysis with p53S367p antibody, high levels of p53S367p were detected in undamaged control cells, but its marked decrease was evident 24 h after etoposide/doxorubicin treatment (p53S367p, Eto, and Dox). Because p53S367p was almost completely disappeared in VprBP-depleted cells, and because p53 protein levels were increased after VprBP knockdown, VprBP seems to directly participate in generating p53S367p and destabilizing p53 in U2OS cells. Moreover, the fact that p53S367p and p53 protein levels returned to original levels after the expression of VprBP wild-type, but not VprBPK194R kinase-dead mutant, in VprBP-depleted U2OS cells (Fig. 2A, Supplementary Fig. S2A) strongly argues that VprBP kinase activity is critical for modulating the S367p and stability of p53. The treatment of U2OS cells with VprBP inhibitor B32B3 at the final concentration of 0.5 µM also led to a significant reduction in p53S367p and showed detectable effects on p53 protein levels following etoposide-induced DNA damage (Eto + B32B3). The observed correlation between p53S367p and p53 protein levels suggests the possibility that p53S367p carries out its negative effects on p53 function by controlling the intrinsic stability of p53.

Our demonstration of VprBP binding to recombinant p53 persuasively support the notion that VprBP-mediated p53S367p requires a stable kinase-substrate docking interaction. However, it is not clear whether VprBP utilizes a similar docking mechanism to phosphorylate p53 in U2OS cells. In approaching this question, lysates from U2OS cells were immunoprecipitated with anti-p53 antibody and subjected to Western blot analysis. Our results showed that p53 can stably associate with VprBP in undamaged control U2OS cells. However, etoposide-induced DNA damage and B32B3 treatment significantly compromised the ability of p53 to associate with VprBP in U2OS cells. We also found that B32B3 treatments have only minimal effects on the observed attenuation of intracellular VprBP-p53 association in etoposide-treated damaged cells (Fig. 2B). In additional analysis, p53 was able to interact with ectopic VprBP wild-type and K194R kinase-dead mutant that were expressed in VprBP-depleted cells (Fig. 2B). It thus seems that, although other factors may be involved, cellular p53S367p by VprBP is likely mediated through a direct substrate docking mechanism.

Since p53 functions as a DNA binding protein that stimulates transcription from downstream target genes, we next wanted to investigate whether VprBP-mediated S367p plays any role in modulating p53 transcriptional activity. Toward this end, we first conducted in vitro transcription assays using a DNA template containing p53 response elements as described [36]. In initial assays, robust activation of transcription was observed with p53 wild-type, and inclusion of VprBP and ATP resulted in no detectable change in p53 transcriptional activity (Supplementary Fig. S3). Likewise, p53S367A mutant was capable of activating transcription with similar efficiency as p53 wild-type regardless of the presence or absence of VprBP and ATP in the transcription reactions (Supplementary Fig. S3). To further investigate the functional impact of VprBP-mediated p53S367p, we checked whether VprBP knockdown exerts any effects on p53 transcription and p53 target gene expression by reverse transcription quantitative PCR (RT-qPCR). Somewhat surprisingly and inconsistent with our expectations from in vitro transcription results, depletion of VprBP generated, albeit to a varying extent, a distinct activation of p53 responsive genes and ectopic expression of VprBP wild-type completely reversed the effects of VprBP knockdown in U2OS cells (Fig. 2C, Supplementary Fig. S2B, S4A). By comparison, no changes in p53 mRNA levels were apparent following VprBP knockdown in U2OS cells (Supplementary Fig. S5). Unlike VprBP wild-type, K194R kinase-dead mutant failed to generate any effects on target gene expression in VprBP-depleted cells (Fig. 2C, Supplementary Fig. S2B, S4A). These results were further corroborated by the inhibitor experiments in which VprBP wild-type failed to restore the original target gene expression levels in VprBP-depleted U2OS cells in the presence of VprBP inhibitor B32B3 (Fig. 2C, Supplementary Fig. S2B, S4A).

Having demonstrated a negative impact of VprBP-mediated S367p on cellular p53 function, ChIP analysis was also performed to check whether the observed effects are directly linked to S367p-induced inhibition of p53 binding to target genes. Cross-linked chromatin was isolated from U2OS cells and sonicated to mono- and di-nucleosomes after cross-linking. The precipitated nucleosomal DNA was extracted and amplified by qPCR using primers specific for p53 response element regions of target genes. Although the precipitation efficiency slightly varied among the target genes, we were able to detect higher p53 ChIP signals in etoposide-treated cells compared to untreated control cells. Under these assay conditions, p53 levels at target genes were elevated after VprBP knockdown, confirming the direct involvement of VprBP in reducing the extent of target gene occupancy by p53 (Fig. 2D, Supplementary Fig. S2C, S4B). When ChIP experiments were performed after the expression of VprBP wild-type or K194R kinase-dead mutant in VprBP-depleted U2OS cells, only VprBP wild-type rescued the effects of VprBP knockdown.

Given that p53 responsive genes are suppressed by VprBP and that p53 regulates cell growth through apoptosis and cell cycle arrest, we also examined whether VprBP-mediated p53S367p influences the growth of U2OS cells. As summarized in Fig. 2E, the ability of undamaged cells to grow was not much affected when VprBP was depleted and rescued (DMSO). However, similar assays under etoposide-induced DNA damage condition showed a distinct decrease in the growth rate of U2OS cells, and VprBP knockdown resulted in more significant drop in cell growth capacity (Eto). In line with the requirement of S367p for VprBP function in stimulating cell growth, the original growth rate of U2OS cells was restored after expressing VprBP wild-type, but not kinase-dead mutant, in VprBP-depleted cells (Fig. 2E). These results, together with our observation that treatment with VprBP inhibitor B32B3 almost completely crippled the rescue effects of ectopic VprBP, support the conclusion that VprBP-mediated p53S367p is critical for VprBP function in promoting the growth of U2OS cells.

### S367p reduces p53 stability and transactivation capacity

**Fig. 3:**
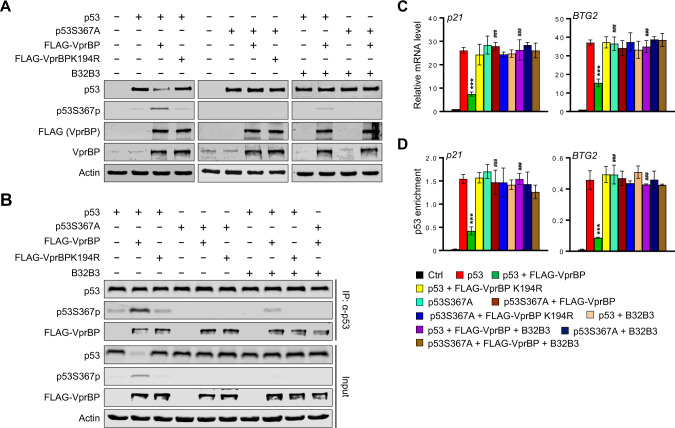
S367p-induced destabilization and inactivation of p53. **A** H1299 cells were transfected with VprBP and/or p53 expression plasmids for 48 h in the presence or absence of B32B3 (3 µM) as indicated on the top. Extracts were prepared in lysis buffer and Western blot analysis was performed with antibodies against p53, p53S367p, FLAG, and VprBP. **B** Whole cell lysates from (**A**) were subjected to immunoprecipitation using p53 DO-1 antibody. The binding of VprBP to p53 was analyzed by Western blotting. **C** Total RNA was isolated from the H1299 cells transfected with VprBP and/or p53 expression plasmids as in (**A**), and subjected to RT-qPCR analysis with p21 and BTG2 specific primers listed in Supplementary Table S1. Data represent the means ± SD of three independent experiments. *P* values were calculated using two-way ANOVA with post-hoc Tukey’s test for multiple comparisons. ***P < 0.001 versus p53; ^###^*P* < 0.001 versus p53 + FLAG-VprBP. (See also Supplementary Fig. S8A). **D** ChIP assays were performed in the H1299 cells transfected with VprBP and/or p53 expression plasmids as in (**A**) using p53 DO-1 antibody. Precipitated DNA was amplified with specific primers for p21 and BTG2 listed in Supplementary Table S2. Data are represented as mean ± SD of three independent experiments. *P* values were calculated using two-way ANOVA with post-hoc Tukey’s test for multiple comparisons. ***P < 0.001 versus p53; ^###^*P* < 0.001 versus p53 + FLAG-VprBP. (See also Supplementary Fig. S8B).

As an extension of the above-described studies suggesting an inverse relationship between p53S367p and p53 function, it was important to evaluate the effects of VprBP-mediated S367p on p53-dependent pathway more directly. For this objective, we decided to use H1299 human lung cancer and T84 human colon cancer cells which do not express p53 and contain nearly undetectable levels of VprBP. In keeping with the data from U2OS cells (Fig. 2A), our Western blot analysis using the p53S367p antibody showed that p53S367 is phosphorylated in H1299 and T84 cells co-transfected with p53 and VprBP (Fig. 3A, Supplementary Fig. S6A, lane 3). Also, confirming the specificity of the p53S367p antibody and the VprBP target phosphorylation site, no p53S367p was detected in H1299 and T84 cells expressing p53S367A mutant and VprBP (lanes 5-8). The fact that VprBPK194R kinase-dead mutant was not capable of generating p53S367p (lane 4) strongly argues that VprBP is responsible for mediating the observed p53S367p. To determine whether S367p has a role in modulating p53 stability, we also compared the steady-state level of p53 wild-type to that of p53S367A mutant. Remarkably, combined expression of p53 wild-type and VprBP in H1299 and T84 cells resulted in a marked reduction in the p53 protein levels (lanes 2 and 3). The observed change was dependent of S367p status, as S367A phosphorylation-blocking mutation significantly compromised the p53-reducing effects of VprBP (lanes 3 and 7). Moreover, exposure to VprBP inhibitor B32B3 (lanes 9-12) or expression of VprBPK194R kinase-dead mutant (lane 4) restored p53 steady levels in H1299 and T84 cells, thus again implying that VprBP regulates p53 stability through its phosphorylation of S367.

Since phosphorylation often acts as a signal to modulate protein stability, we also treated H1299 cells expressing p53 wild-type or S367A mutant with the protein synthesis inhibitor cycloheximide (CHX) for 2 h, and evaluated its impact on p53 protein levels by Western blotting. Intriguingly, our analysis detected an equivalent level of p53S367A mutant protein, but a fast disappearance of p53 wild-type protein, in CHX-treated H1299 cells (Supplementary Fig. S7A), an indicative of a possible role of VprBP-mediated S367p in decreasing p53 protein stability. Additionally, that treating p53-transfected H1299 cells with the proteasome inhibitor MG132 generated an apparent increase in p53 proteins levels argues strongly that the observed effects of VprBP-mediated S367p on p53 reflects an alteration of proteomic degradation processes (Supplementary Fig. S7B). To further understand the p53-stablizing process, the interaction of p53 with VprBP was tested by Western blot analysis of anti-p53 immunoprecipitates from lysates of H1299 cells that were transfected with combinations of plasmids encoding p53 and VprBP. As shown in Fig. 3B, when equal amounts of immunoprecipitated p53 were subjected to Western blotting, we found that p53 can generate a stable interaction with both VprBP wild-type and K194R kinase-dead mutant (lanes 2 and 3). In agreement with our observation in U2OS cells, additional binding experiments in which co-immunoprecipitation was carried out using lysates of p53S367A mutant-transfected cells disclosed that VprBP can interact with p53S367A mutant with affinity similar to that of p53 wild-type (Fig. 3B, lanes 2 and 5). Meanwhile, we found that B32B3 is unable to impact p53-VprBP interaction in H1299 cells (lanes 7-10), results supportive of the view that altered VprBP binding properties of p53 are not part of the mechanisms by which VprBP-mediated S367p regulates p53 cellular activity.

To assess the functional contributions made by p53S367p in the above results, we conducted RT-qPCR analysis of the four representative p53 target genes. The expression of ectopic p53 activated target gene transcription in H1299 and T84 cells, but co-expression of VprBP significantly reduced the transcription levels of p53 target genes (Fig. 3C, Supplementary Figs. S6B, S8A). In checking the effects of p53S367p, VprBPK194R kinase-dead mutant failed to diminish the extent of target gene induction in p53-transfected cells. Consistent with the concept of p53S367p as an essential process for VprBP function, we also noticed that VprBP is not effective in inhibiting p53 target gene transcription in H1299 and T84 cells expressing p53S367A phosphorylation-blocking mutant (Fig. 3C, Supplementary Figs. S6B, S8A). These results are in complete agreement with data indicating that treatment with VprBP inhibitor B32B3 could reactivate p53 target genes in H1299 and T84 cells expressing p53 and VprBP wild-type, but not p53S367A and VprBPK194R mutant.

In accordance with our RT-qPCR data, ChIP experiments using p53 antibody showed a stable occupancy of the response element regions of target genes by ectopic p53 in H1299 and T84 cells. On the contrary, when p53 was co-expressed with VprBP, the observed p53 enrichment in target genes was compromised, as indicated by a significant reduction in p53 ChIP signal (Fig. 3D, Supplementary Figs. S6C, S8B). Because VprBP regulates p53 function through its kinase activity, we also checked the levels of p53 at target genes after replacing VprBP wild-type with VprBPK194R kinase-dead mutant. Our analysis clearly demonstrated that target gene occupancy of p53 was not affected by VprBPK194R mutant in H1299 and T84 cells (Fig. 3D, Supplementary Figs. S6C, S8B). These results raised another question of whether the negative impact of VprBP on p53 enrichment at target genes is dependent on S367p. In fact, the predicted role for VprBP-mediated S367p is confirmed by the observation that p53 ChIP signal in p53S367A mutant-expressing cells was about 3-5 times higher compared to those obtained with p53S367 wild-type-expressing cells (Fig. 3D, Supplementary Figs. S6C, S8B). These observations were further corroborated by additional ChIP assays showing that chromatin prepared from B32B3-treated, VprBP-transfected cells generates a higher p53 ChIP signal relative to untreated VprBP-transfected cells (Fig. 3D, Supplementary Figs. S6C, S8B).

### p53 acetylation prevents its interaction with VprBP and S367p-induced destabilization

**Fig. 4:**
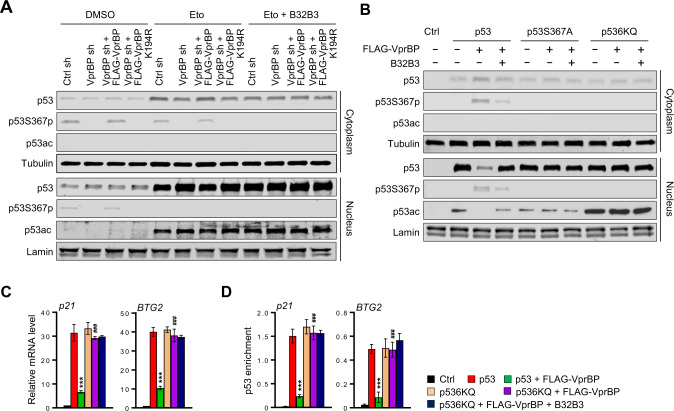
Nuclear p53 stability and activity modulated by S367p and acetylation. **A** Control, VprBP-depleted, VprBP/VprBPK194R-rescued U2OS cells were treated with etoposide (40 µM) in the presence or absence of B32B3 (0.5 µM) for 24 h. Cytoplasmic extracts, and nuclear extracts were prepared and analyzed by Western blotting with antibodies against p53, p53S367p and p53ac. **B** H1299 cells were transfected with FLAG-VprBP and/or p53, p53S367A or p536KQ for 48 h in the presence or absence of B32B3 (3 µM). Cytoplasmic extracts and nuclear extracts were prepared and analyzed by Western blotting with indicated antibodies. **C** Total RNA was isolated from the H1299 cells transfected with VprBP/p53 expression plasmids as in (**B**), and subjected to RT-qPCR analysis with p21 and BTG2 specific primers listed in Supplementary Table S1. Data represent the means ± SD of three independent experiments. *P* values were calculated using two-way ANOVA with post-hoc Tukey’s test for multiple comparisons. ***P < 0.001 versus p53; ^###^*P* < 0.001 versus p53 + FLAG-VprBP. (See also Supplementary Fig. S10A). **D** ChIP assays were performed in the H1299 cells transfected with VprBP/p53 expression plasmids as in (**B**) using p53 DO-1 antibody. Precipitated DNA was amplified with specific primers for p21 and BTG2 listed in Supplementary Table S2. Data are represented as mean ± SD of three independent experiments. *P* values were calculated using two-way ANOVA with post-hoc Tukey’s test for multiple comparisons. ***P < 0.001 versus p53; ^###^*P* < 0.001 versus p53 + FLAG-VprBP. (See also Supplementary Fig. S10B).

Because p53 acetylation at the C-terminal regulatory domain promotes p53 stability and function in response to DNA damage, our finding that VprBP drives p53 degradation suggests that p53 acetylation may be vital to the control of VprBP-mediated p53S367p in damaged cell nuclei. To check this possibility, cytoplasmic and nuclear fractions were prepared from untreated and etoposide-treated U2OS cell extracts, and their equivalent proportions were probed with p53-K373ac/K382ac antibody by Western blotting. A noteworthy observation emerged from these experiments was that the acetylation levels of p53 were significantly enhanced in the nucleus, while acetylated p53 was not detectable in the cytoplasm, 24 h following etoposide treatment (Fig. 4A, DMSO and Eto). Also, no detectable changes in p53 acetylation levels after VprBP knockdown and rescue expression indicate that p53 acetylation is not affected by VprBP-mediated S367p (Eto). Consistent with these results, treatment of U2OS cells with VprBP inhibitor B32B3 failed to show any effects on p53 acetylation in response to etoposide-induced DNA damage (Eto + B32B3).

As a more direct approach to study the role of p53 acetylation in controlling VprBP-mediated S367p, we also analyzed nuclear and cytoplasmic fractions that were prepared from H1299 cells expressing ectopic p53 and VprBP. The results correlated well with those obtained from etoposide-treated U2OS cells and demonstrated that the steady-state level of acetylated p53 was maintained mainly in the nuclei of H1299 cells expressing p53 wild-type (Fig. 4B, Supplementary S9A, p53). Moreover, the finding that the acetylation status of p53 was not affected by VprBP expression and S367D mutation reconfirmed that p53 acetylation acts as an upstream regulator of VprBP-mediated p53S367p (Supplementary Fig. S9A, B). To further support these results, our analysis was repeated with nuclear and cytoplasmic fractions of H1299 cells transfected with p536KQ mutant mimicking constitutive acetylation. Unexpectedly, we found that the p53ac antibody used in our assays cross-reacted with ectopic p536KQ mutant and generated a nonspecific band in our analyses (p536KQ). Whereas similar cross-reactivity was observed with a p53ac antibody from another source (Abcam), these commercial p53ac antibodies were not reactive against p536KR and other p53 mutants (not shown), supporting the specificity of the antibodies. As shown in Fig. 4B, our studies with p53 and p53S367p antibodies revealed a distinct increase in p53 protein levels and an almost complete loss of p53S367p after the expression of p536KQ acetylation mimicking mutant (p536KQ). Along with these p536KQ mutant results, the fact that p536KR acetylation blocking mutant displays a stable accumulation to high levels in the cytoplasm clearly supports the view that p53 acetylation could attenuate S367p-induced p53 degradation (Supplementary Fig. S9A). The observed effects of p53 mutations were dependent of proteomic degradation process, because those mutations generated little to no changes in p53 protein levels in MG132-treated H1299 cells (Supplementary Fig. S9B).

Since the observed changes in p53 protein and S367p levels after 6KQ acetylation mimicking mutations might be functionally significant, we also examined the transcriptional states of target genes. The results of these efforts showed that VprBP is not capable of suppressing the expression of p53 target genes in p536KQ mutant-transfected cells (Fig. 4C, Supplementary Fig. S10A). Congruent with these data, when ChIP assays were conducted using H1299 cells transiently transfected with p536KQ mutant, a larger accumulation of p536KQ mutant around response element regions was observed as compared with p53 wild-type (Fig. 4D, Supplementary Fig. S10B). To further confirm these results and evaluate the effects of p53 acetylation in other cell types, we repeated Western blot analysis and RT/ChIP-qPCR assays with T84 colon cancer cells; similar results were obtained from these parallel experiments (Supplementary Fig. S11). Collectively, these observations are consistent with those of earlier studies of p53-VprBP interaction [36, 39] and establish p53 acetylation as a critical regulatory mechanism for direct p53 association with VprBP in cellular environments.

### p53 nuclear export and proteasomal degradation are regulated by S367p and acetylation

**Fig. 5:**
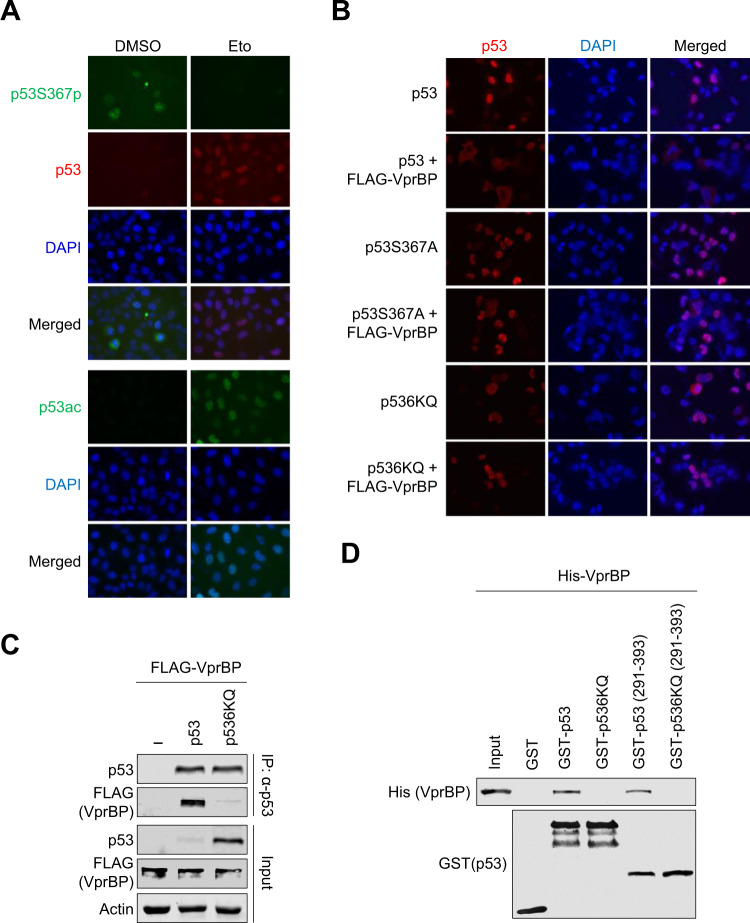
p53-VprBP interaction and p53 nuclear export regulated by S367p and acetylation. **A** U2OS cells were treated with DMSO and etoposide (40 µM) for 24 h and immunostained with p53S367p, p53 and p53ac antibodies. **B** H1299 cells were transfected with VprBP and/or p53 expression plasmids for 48 h as indicated on the left and immunostained with anti-p53 antibody. **C** H1299 cells were transfected with FLAG-VprBP and/or p53 or p536KQ for 48 h. Extracts were prepared in lysis buffer and were subjected to immunoprecipitation using p53 DO-1 antibody. The binding of VprBP to p53 was analyzed by Western blotting. **D** GST alone or the indicated GST‐p53 fusions were immobilized on glutathione‐Sepharose beads and incubated with His‐VprBP. After washing, bound VprBP proteins were fractionated by SDS/PAGE and examined by Western blotting with anti‐His antibody.

Overall, the knockdown and overexpression studies described above indicate that p53 nuclear accumulation and function were sequestered by VprBP-mediated S367p but enhanced by acetylation. In order to gain further support for such opposing effects of p53S367p and acetylation, untreated or etoposide-treated U2OS cells were examined by immunostaining. As seen in Fig. 5A, p53S367p was localized predominantly in the cytoplasm of untreated U2OS cells (upper left). Meanwhile, when cells were treated etoposide, p53S367p was detected in the nuclei at very low levels (upper right). In marked contrast, immunostaining analyses using p53 and p53-K373ac/K382ac antibodies reproducibly showed that etoposide treatment of U2OS cells leads to a significant increase in the levels of p53 and its acetylation in the nucleus (lower). Likewise, H1299 cells expressing p53 in the absence of VprBP exhibited predominantly nuclear staining, but the co-expression of VprBP wild-type stimulated the cytoplasmic translocation of p53 (Fig. 5B, Supplementary S12). It was also apparent that such a nuclear to cytoplasmic movement of p53 was not observed when p53S367A mutant was used in our experiments. This observation strongly argues for an important role of VprBP-mediated p53S367p in regulating p53 subcellular localization and is further corroborated by the experiments using p53S367D phospho-mimicking mutant which is continuously degraded and maintained at a low level (Supplementary Fig. S9). Additionally, we observed that a major portion of p536KQ acetylation-mimicking mutant is present in the nucleus and its staining intensity is unaffected upon co-expression of VprBP (Fig. 5B).

A direct interaction between p53 and VprBP was identified as being critical for VprBP kinase activity toward p53 in our investigation. Thus, a plausible explanation for the observed effects of p53 acetylation is that it could perturb p53-VprBP interaction. To investigate this possibility, we co-transfected H1299 cells with either p53 wild-type or 6KQ mutant and VprBP expression vectors, and immunoprecipitated ectopic p53 from whole-cell lysates with p53 antibody. As is apparent from Fig. 5C, p53 antibody was able to co-immunoprecipitate VprBP from lysates of H1299 cells expressing p53 wild-type. In an attempt to further substantiate these binding data, we then conducted GST pull-down assays using GST-fused p53 and His-tagged VprBP. Both p53 full length and C-terminal domain (residues 291-393) showed a comparable interaction with VprBP, as determined by Western analysis of the binding reactions (Fig. 5D). The observed interaction was largely compromised upon 6KQ acetylation-mimicking mutation of p53 C-terminal region, and these results strongly suggest that acetylation promotes p53 stability and nuclear accumulation by disrupting p53 binding to VprBP and thus VprBP-mediated p53S367p.

**Fig. 6:**
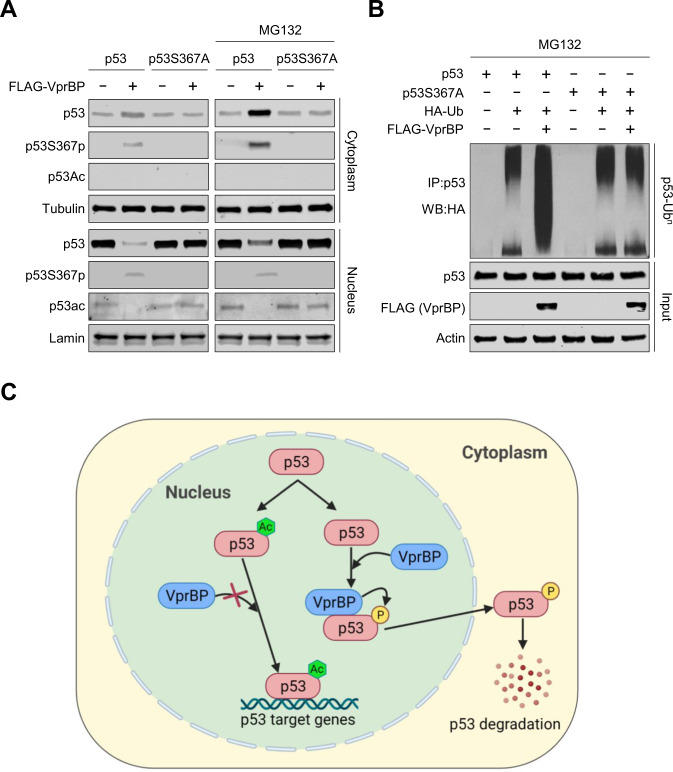
Targeting p53 for ubiquitination and proteasomal degradation by S367p. **A** H1299 cells were transfected with FLAG-VprBP and/or p53 or p53S367A for 48 h. 6 h before the end of the experiment, 20 µM MG132 is added. Cytoplasmic and nuclear extracts were prepared and analyzed by Western blotting with antibodies against p53, p53S367p and p53ac. **B** H1299 cells were transfected as in (A) but in presence or absence of vector expressing HA-Ub for 48 h. 20 µM MG132 is added for 6 h before harvesting cells. Extracts were prepared in lysis buffer and were subjected to immunoprecipitation using p53 DO-1 antibody followed by Western blotting with anti-HA antibody. **C** Model representing VprBP-mediated p53S367p and degradation.

As p53 is mainly degraded through the ubiquitin–proteasome mechanism, we hypothesized that VprBP-mediated S367p may affect the rate of p53 degradation process in the cytoplasm. In order to test this possibility, we treated H1299 cells with the proteasome inhibitor MG132 followed by preparation of cytoplasmic and nuclear fractions and performed Western analysis for p53, p53S367p, and p53ac. This approach showed that MG132 treatment increased steady-state levels of p53 and p53-S376p in the cytoplasmic fraction of H1299 cells expressing p53 wild-type and VprBP, whereas there was no change in their levels in the nuclear fraction (Fig. 6A). Opposite of p53 wild-type, p53S367A mutant was abundant in the nuclei of MG132-treated H1299 cells and less concentrated in the cytoplasm. In further agreement, MG132 treatment did not induce any changes in p53 acetylation, consistent with the idea that p53S367p is sufficient to induce the cytoplasmic localization and degradation of p53 via the ubiquitin-proteasome pathway. We then transfected hemagglutinin (HA)-tagged ubiquitin in H1299 cells expressing p53/VprBP and immunoprecipitated ubiquitinated p53 in the presence of MG132. In checking the levels of polyubiquitinated p53 proteins, we observed that VprBP wild-type, but not VprBPK194R kinase-dead mutant, is able to induce an increase in p53 polyubiquitination in MG132-treated H1299 cells transfected with p53 wild-type (Fig. 6B and S13). Conversely, there were almost no detectable effects of VprBP on the polyubiquitination levels of p53S367A mutant in MG132-treated H1299 cells, all in all implying that p53S367p plays a role in facilitating p53 destabilization and degradation.

## Discussion

Our recent reports identified VprBP as being functional in repressing p53 transcriptional activity and more importantly having an intrinsic kinase activity to promote histone H3 phosphorylation and gene silencing [36, 37]. As the involvement of VprBP in cancer phenotypes becomes increasingly evident, examining other potential phosphorylation targets of VprBP besides H3 is a logical way to fully understand the mechanisms underlying VprBP-induced tumorigenesis. In the work described here, we investigated the molecular basis for VprBP function in attenuating p53 tumor suppressor pathway and accelerating cancer development. Our initial analysis demonstrated that VprBP can directly interact with p53 and catalyze phosphorylation in the carboxyl-terminal regulatory domain of p53. The mass spectrometric identification of S367 as a major phosphorylation site of p53 and development of p53S367p-specific antibody allowed us to confirm that p53S367p takes place in a VprBP-dependent manner. Also, protein-protein interaction assays provided evidence that VprBP-mediated p53S367p event requires the physical binding of p53 to VprBP. Our knockdown and overexpression studies further indicated that p53S367p by VprBP is responsible for keeping p53 target genes inactive and consequently generating defects in DNA damage response. In an understanding of the underlying mechanism, we found that VprBP-mediated S367p makes p53 to become more susceptible to proteasomal degradation. As such, a reduced level of p53S367p is coincident with an increased stability of p53 after DNA damage, reinforcing the notion that VprBP-mediated p53S367p suppresses target gene expression by negatively regulating p53 stability. The importance of VprBP-mediated S367p per se in triggering p53 degradation through proteasome pathway was also illustrated by our demonstration that p53 protein levels were significantly increased after S367A mutation. Furthermore, VprBP-mediated p53S367p renders cells resistant to DNA damage-induced apoptosis, suggesting that maintaining low levels of p53S367p is important for p53 to trigger apoptosis in response to DNA damage. The functional importance of blocking S367p in p53 transactivation is also supported by the observation that p53S367A phosphorylation defective mutant is capable of inducing the expression of p53 response genes when co-transfected with VprBP. Importantly, the observed impairment of p53 function after S367p is not due to the loss of its tetramerization capacity, as reflected by the fact that the tetrameric formation of p53S367A mutant looks very similar to that of the wild-type counterpart in the presence of VprBP. Therefore, it is tempting to speculate that p53 degradation after VprBP-mediated S367p is mainly related to recognizing S367p by a ubiquitin targeting enzyme, thus directing p53 ubiquitination and proteasomal degradation.

Another key finding from our study is that DNA damage-induced acetylation of p53 at carboxyl-terminal lysine residues increases p53 protein levels through attenuation of VprBP-p53 interaction and thus p53S367p. The stable accumulation and transcriptional activity of acetylated p53 at target genes after DNA damage or VprBP inhibitor treatment also fits well to the idea that acetylation plays a crucial role in enhancing p53 protein stability and transcriptional activity. Further supporting these data, we show that acetylation-mimicking mutation of p53 results in a decrease in S367p, which could, in fact, be an indispensable event for enhancing the stability of p53, increasing the recruitment of p53 to target genes, and activating the transcriptional function of p53 upon DNA damage. This finding is intriguing because it supports a model in which p53 stabilization is triggered directly by acetylation, linking p53 hyperacetylation per se to p53 transcriptional activity in response to DNA damage [40–42]. Notably, and consistent with the primary mechanisms of action of p53S367p and p53 acetylation in regulating p53 protein stability, S367A and acetylation-mimicking mutations failed to influence the transcriptional activity of recombinant p53 proteins in our in vitro assays. Defining the molecular basis for how VprBP binding and kinase activity toward p53 is inhibited by p53 acetylation is beyond the scope of this report. However, it would be of particular interest to examine whether the C-terminal acetylation may induce a distinct local structural feature of p53. Related, our previous study showed that VprBP can interact with unmodified histone H3 N-terminal tails, but not with acetylated H3 N-terminal tails, to establish and maintain target genes in an inactive state [36]. In this regard, one obvious possibility is that p53 acetylation acts in an analogous manner to modulate the strength of p53-VprBP interaction, thereby precluding undesired p53S367p as well as leading to p53 nuclear stabilization and transactivation. Although functional relevance of each specific lysine acetylation in p53 C-terminal domain in disabling p53 from interacting with VprBP remains to be determined, it seems clear that p53 acetylation allows for very tight regulation of VprBP-induced disturbance of p53 protein stability and transcriptional activity. Since the expression levels of VprBP are much higher in cancer cells with respect to those in normal cells, our results are also of significance in relation to the key role of VprBP in disrupting p53 signaling pathway during cancer development. Besides acetylation, other types of posttranslational modifications such as ubiquitination and methylation are known to influence p53 activity contributing toward cell cycle arrest and DNA repair [15, 43]. Thus, an additional interesting question to be addressed in future studies should concern a cooperative or antagonistic action of those modifications in controlling VprBP-mediated p53S367p in damaged cells. Apart from the possible contribution of other modifications, specific phosphatases might also participate in reversing the effects of VprBP-mediated p53S367p and initiating DNA damage-induced upregulation and functional activation of p53. Discerning whether this is the case and how S367p deposited by VprBP is selectively removed by phosphatases will help define whether a distinct mechanism is linked to the regulation of DNA damage response at the level of p53 protein stability.

In light of our data presented here, at least two VprBP-dependent events appear to be associated with the control of p53 protein stability, VprBP-mediated S367p, which promotes p53 degradation, and C-terminal acetylation, which protects p53 from degradation (Fig. 6C). Upon DNA damage, the acetylation overrides p53S367p and proteasomal degradation by preventing p53-VprBP interaction. It is not surprising that such a tightly regulated process has evolved for the fine-tuning of tumor suppressive functions of p53 and oncogenic activities of VprBP in response to DNA damage. Therefore, structural investigations of VprBP-bound p53 will provide information on the nature of p53-VprBP interaction and how p53S367p and acetylation regulate p53 signaling pathways. Moreover, the finding that VprBP-mediated p53S367p serves as a crucial step for p53 degradation helps us to develop the strategy of how VprBP can be pharmacologically targeted for the reactivation of p53 in cancer cells.

## Materials and methods

### Kinase assay

In vitro phosphorylation assays were performed with recombinant p53 and VprBP wild-type/mutant in kinase buffer (50 mM Tris-HCl [pH 7.5], 20 mM EGTA, 10 mM MgCl_2_, 1 mM DTT, and 1 mM β-glycerophosphate) containing 10 µCi of [γ-^32^P] ATP (PerkinElmer, Waltham, MA, USA) and 4 mM ATP as recently described [37]. Following incubation at 30 °C for 30 min, p53 and VprBP proteins from each reaction were separated by 5–20% SDS-PAGE, and phosphorylated p53 proteins were visualized by autoradiography.

### RT-qPCR

Total RNA was isolated from U2OS, H1299 and T84 cells using a RNeasy Mini kit (Qiagen, Hilden, Germany) and converted to first-strand cDNA using the SuperScript III First-Strand System Kit (Thermo Fisher Scientific, Waltham, MA, USA). Real-time RT-PCR was carried out with SYBR Green Real-time PCR Master Mixes (Thermo Fisher Scientific, Waltham, MA, USA) according to the manufacturer’s protocol. The primers used for RT-qPCR are listed in Supplementary Table S1. All reactions were run in triplicate, and results were normalized to β-actin mRNA levels.

### ChIP-qPCR

Chromatin immunoprecipitation (ChIP) assays with U2OS, H1299 and T84 cells were performed using the ChIP Assay Kit (Millipore Sigma, St. Louis, MO, USA) as recently described [44]. After reversing the protein–DNA cross-links, immunoprecipitated DNA was purified and analyzed by quantitative real-time PCR (qPCR) using the primers that amplify p53 response element region of p21, BTG2, Reprimo and PUMA genes (Supplementary Table S2). Specificity of amplification was determined by melting curve analysis, and all samples were run in triplicate.

## Supplementary information

Supplementary Figures

Supplementary Tables

Supplementary Figure Legends

Supplementary Experimental Procedures

## Data Availability

All relevant data are available from the authors upon request.
