# Supplementary material for: VprBP/DCAF1 regulates p53 function and stability through site-specific phosphorylation
Source: Oncogene. 2023 Apr 11;42(17):1405–16. doi: 10.1038/s41388-023-02685-8 (PMC10121470; doi:10.1038/s41388-023-02685-8)

Supplementary Figure S1

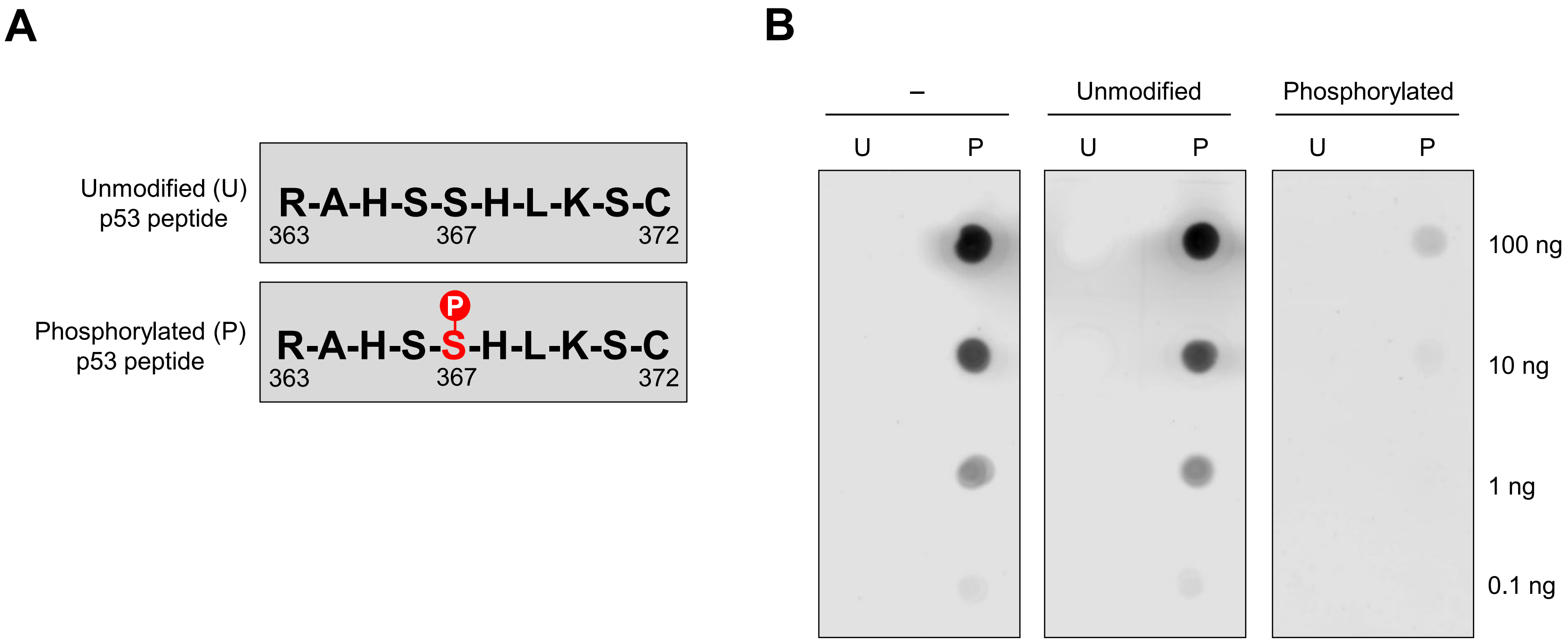

Supplementary Figure S2

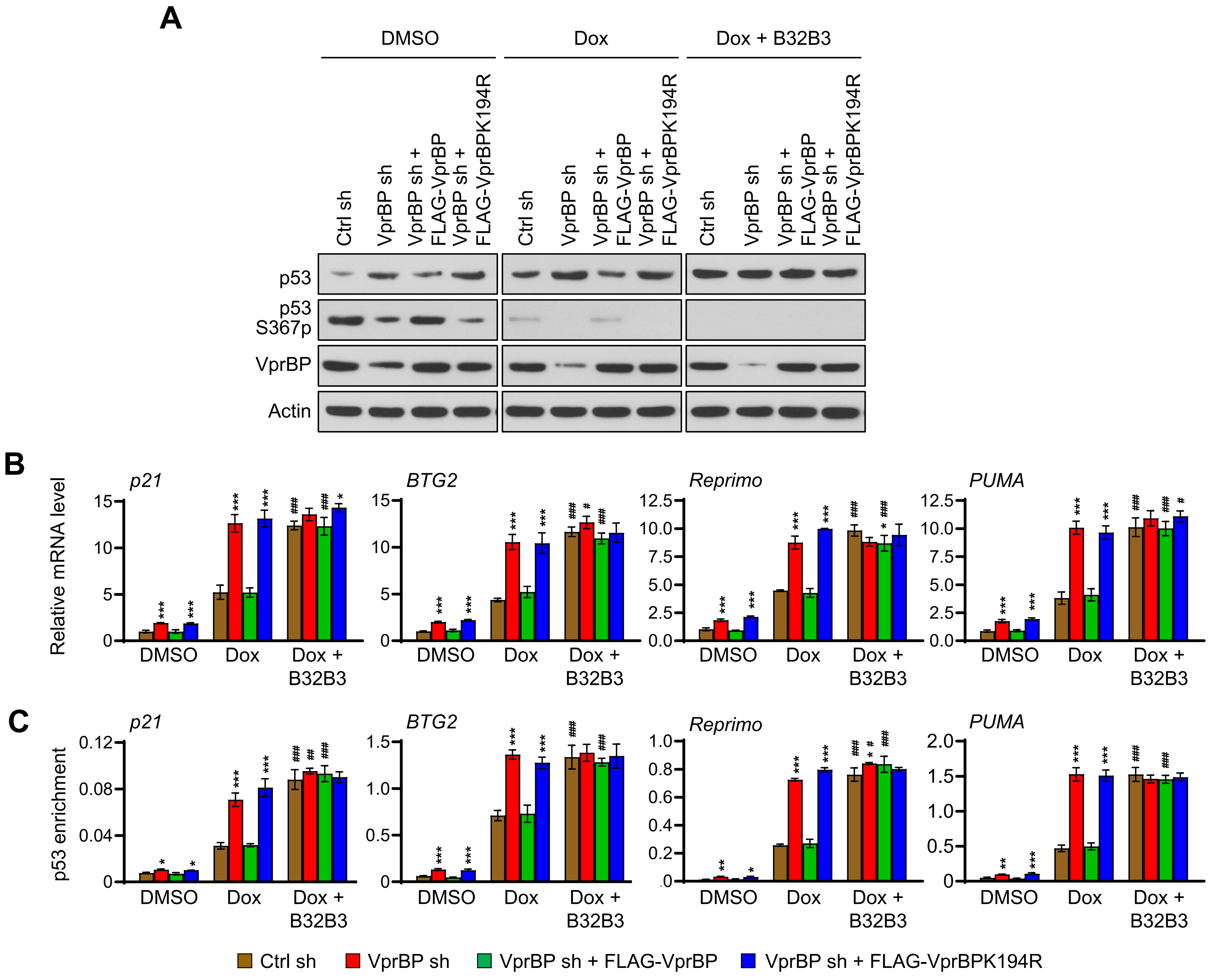

Supplementary Figure S3

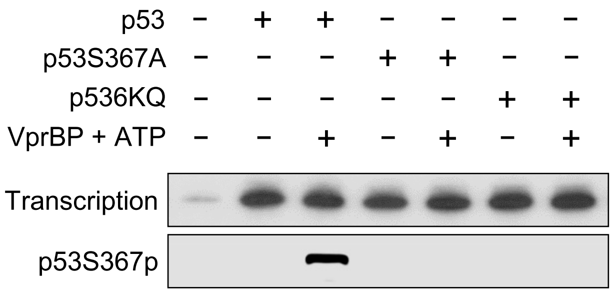

Supplementary Figure S4

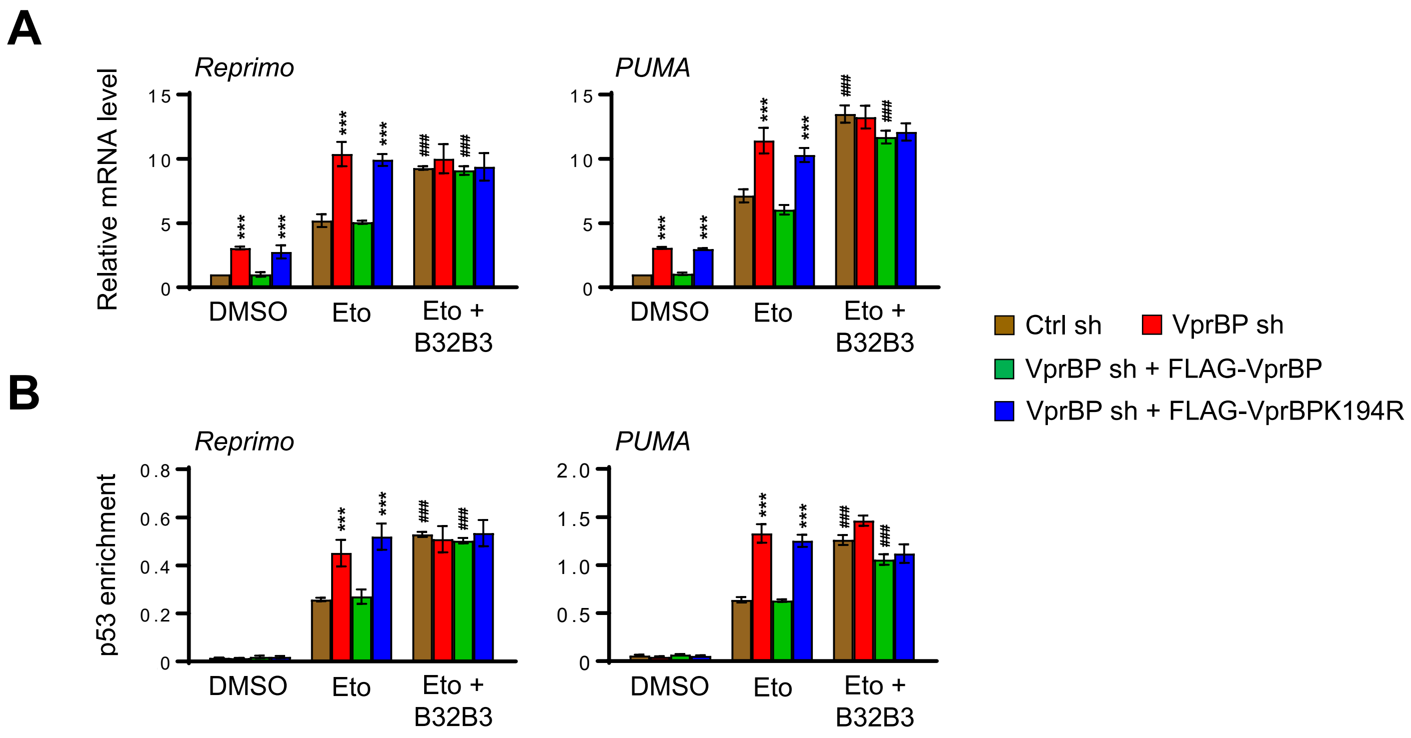

Supplementary Figure S5

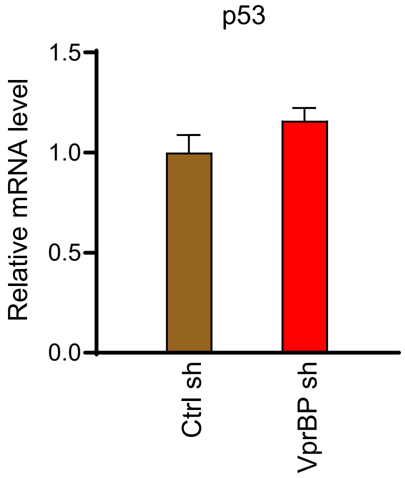

Supplementary Figure S6

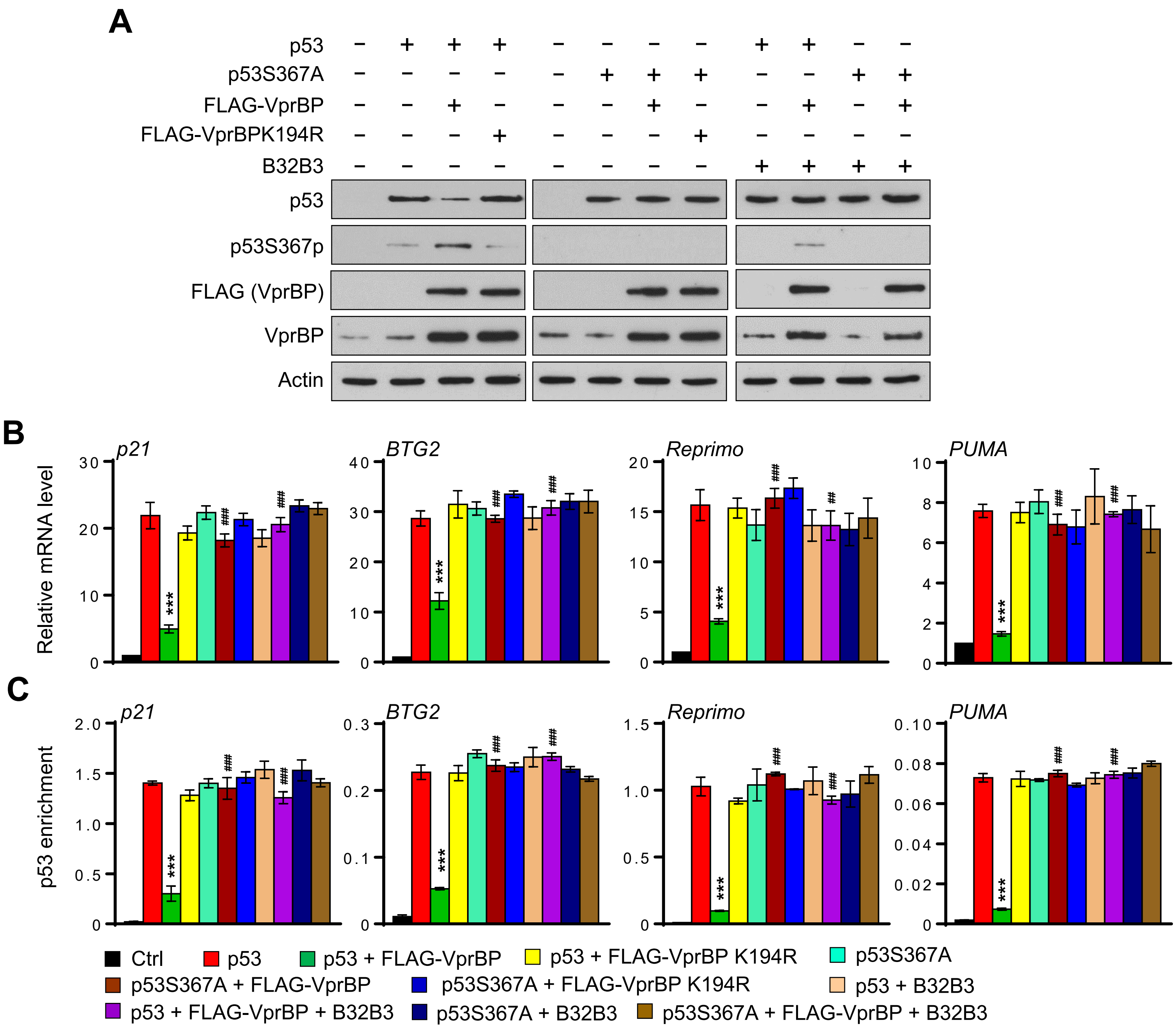

Supplementary Figure S7

A

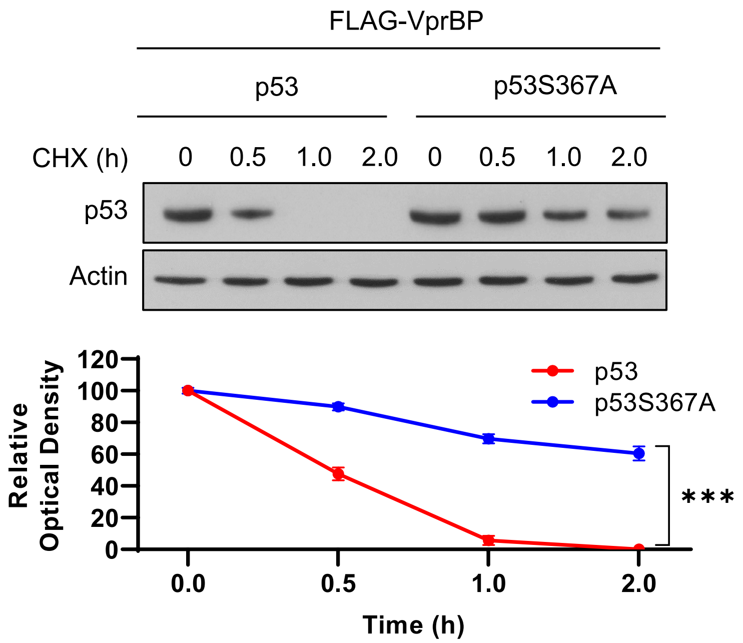

B

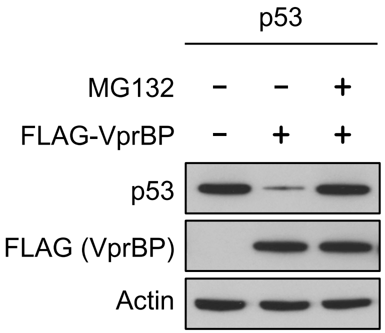

Supplementary Figure S8

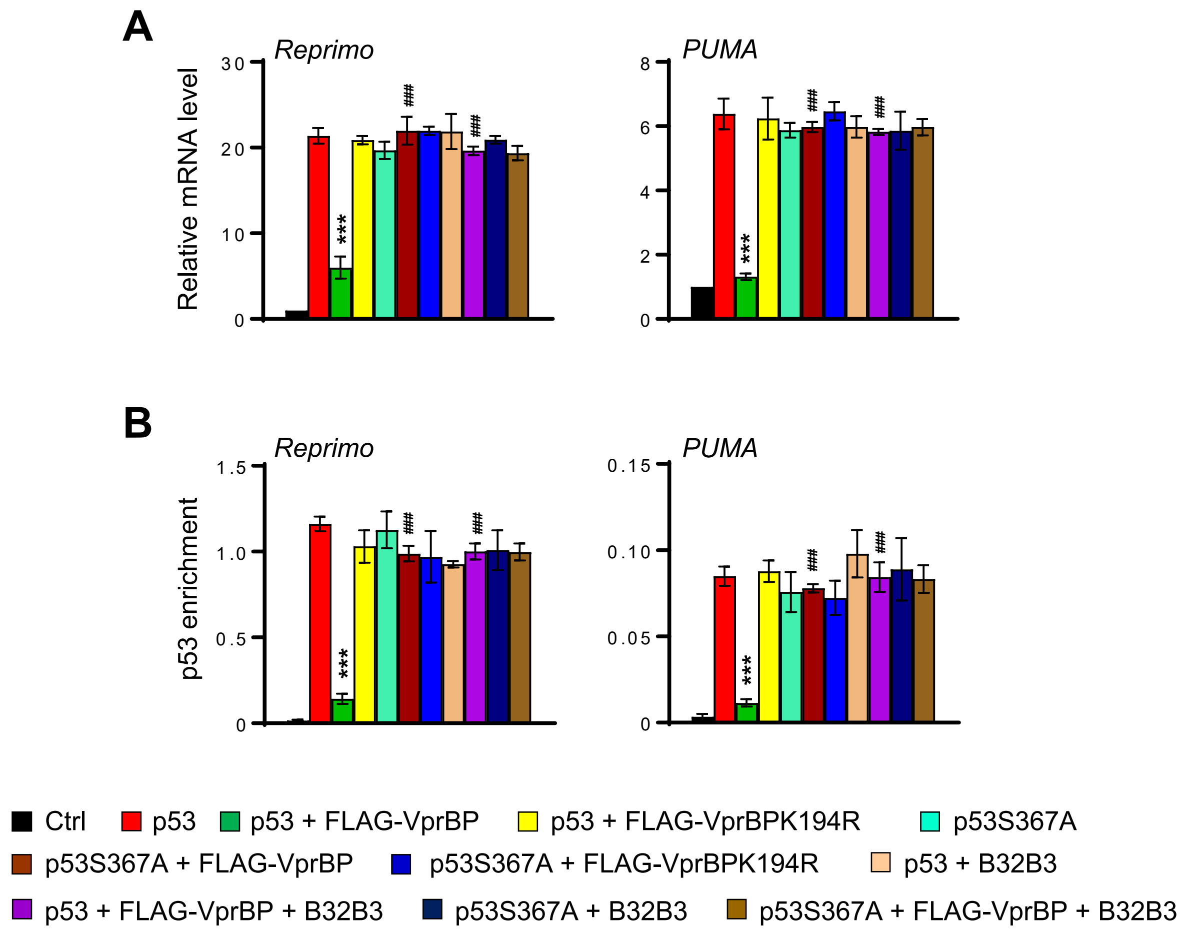

Supplementary Figure S9

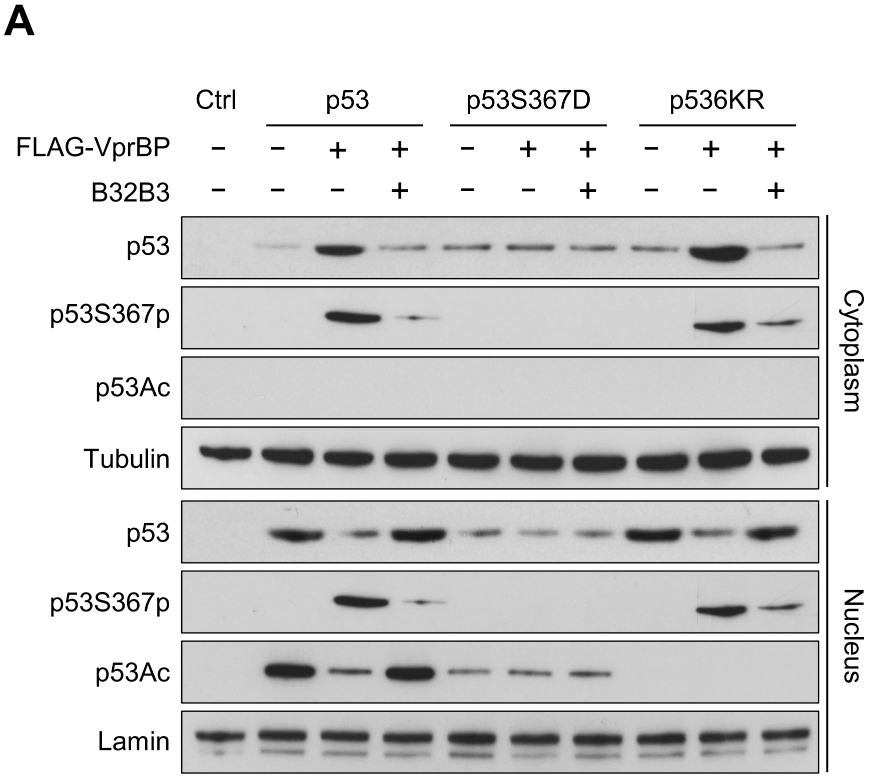

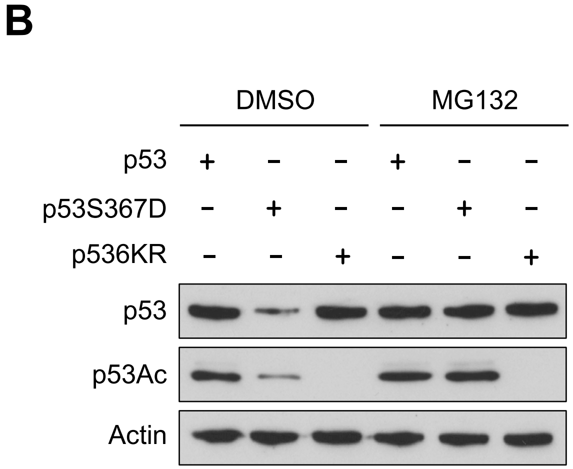

Supplementary Figure S10

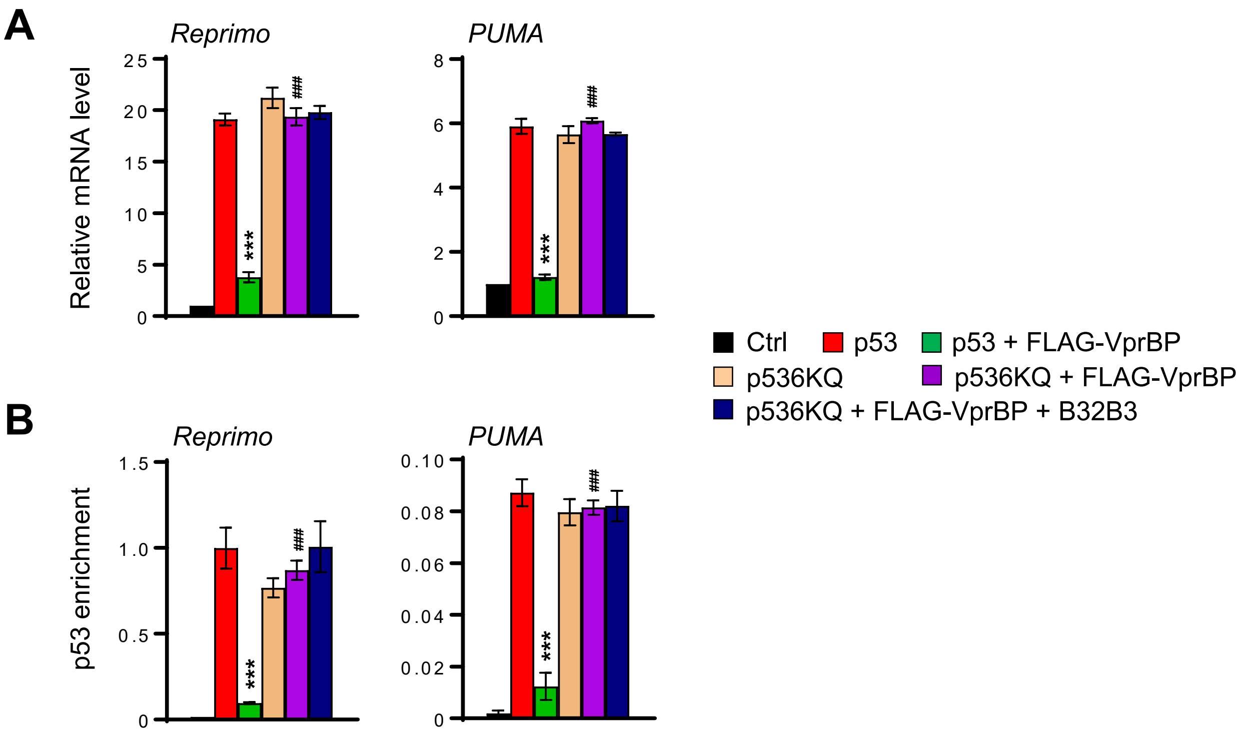

Supplementary Figure S11

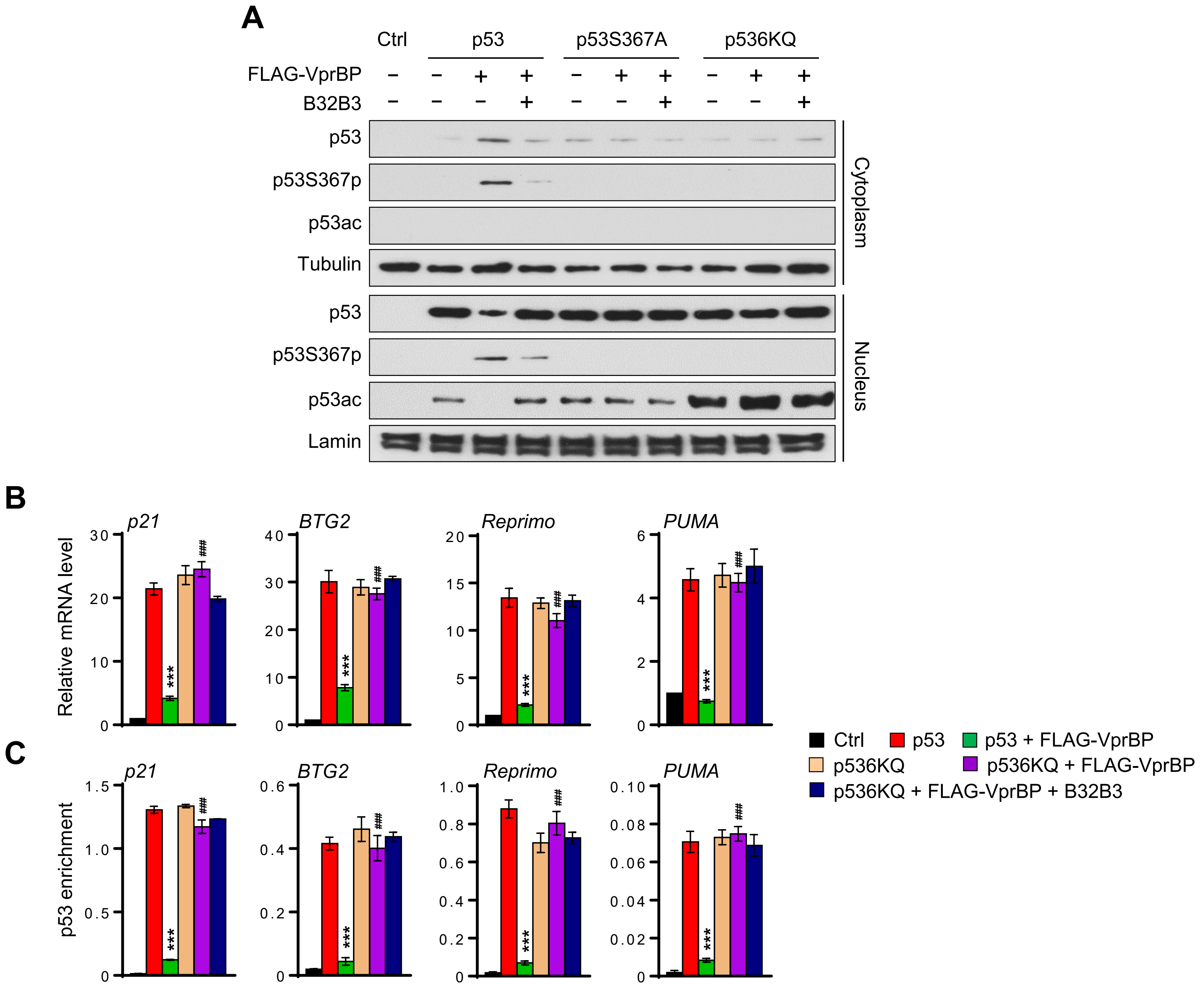

Supplementary Figure S12

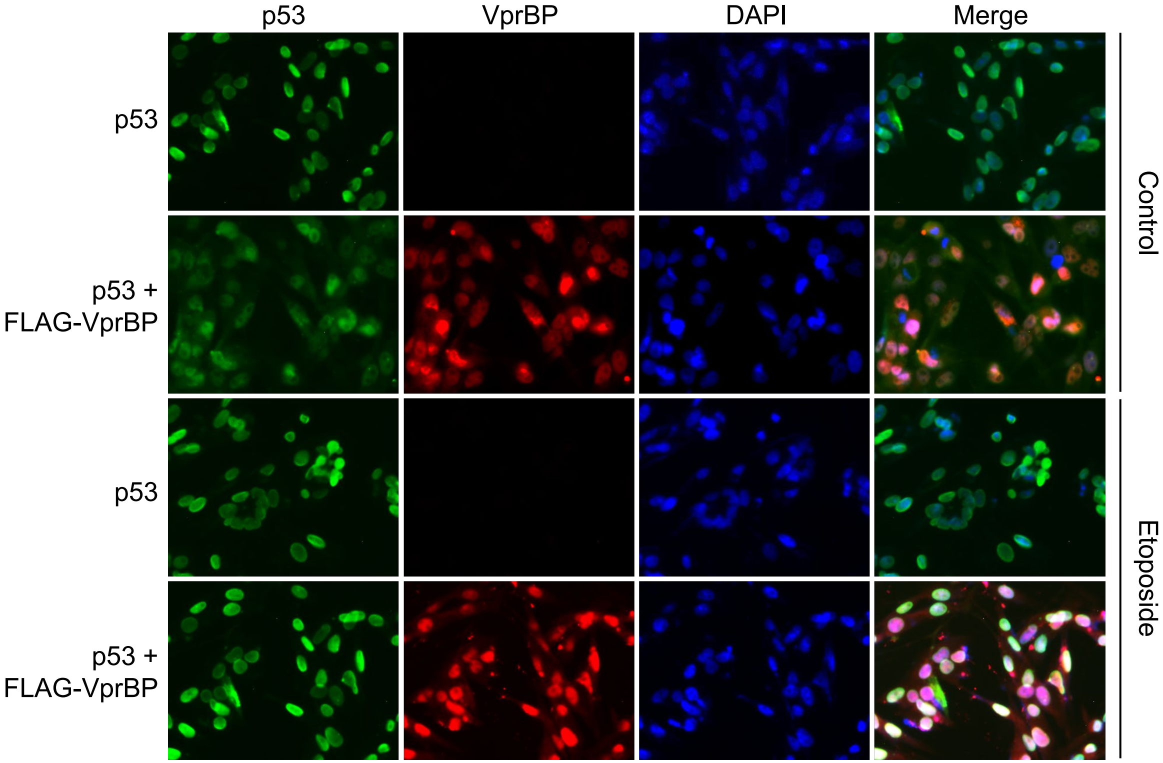

Supplementary Figure S13

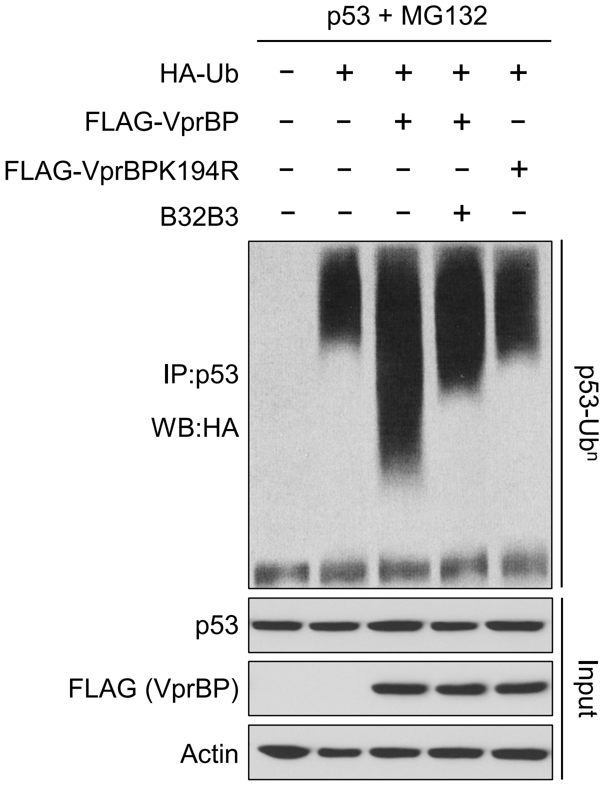

Supplement: Supplementary file 1 — Supplementary Figures [file 41388_2023_2685_MOESM1_ESM.pdf]
