# Supplementary material for: VprBP/DCAF1 regulates p53 function and stability through site-specific phosphorylation
Source: Oncogene. 2023 Apr 11;42(17):1405–16. doi: 10.1038/s41388-023-02685-8 (PMC10121470; doi:10.1038/s41388-023-02685-8)
Supplement: Supplementary file 2 — Supplementary Tables [file 41388_2023_2685_MOESM2_ESM.docx]

**SUPPLEMENTARY INFORMATION**

**Table S1. List of the primers used in RT-qPCR**

| **Gene Name** | **Forward (5’-3’)** | **Reverse primer (5’-3’)** |
| --- | --- | --- |
| p53 | TGAAGCTCCCAGAATGCCAG | GCTGCCCTGGTAGGTTTTCT |
| p21 | ATGGAACTTCGACTTTGTCAC | AGGCACAAGGGTACAAGACAGT |
| BTG2 | TGAGGTGTCCTACCGCATTG | GCACTTGGTTCTTGCAGGTG |
| Reprimo | CTGGCCCTGGGACAAAGAC | TCAAAACGGTGTCACGGATGT |
| PUMA | ACGACCTCAACGCACAGTACGA | GTAAGGGCAGGAGTCCCATGATGA |
| β-actin | GTGGGGCGCCCCAGGCACCA | CTCCTTAATGTCACGCACGATTTC |

**Table S2. List of the primers used in ChIP-qPCR**

| **Gene Name** | **Forward (5’-3’)** | **Reverse primer (5’-3’)** |
| --- | --- | --- |
| p21 | TGGACTGGGCACTCTTGTCC | CAGAGTAACAGGCTAAGGTT |
| Reprimo | GGGGAGGGGCGATAAATACC | GTAACTCCTCAGGCAGGCAA |
| BTG2 | AGACGAGGCAAAGCGGTAAA | TCCAACCATTCACGGTCAGA |
| PUMA | GCGAGACTGTGGCCTTGTGT | CGTTCCAGGGTCCACAAAGT |
