# Supplementary material for: VprBP/DCAF1 regulates p53 function and stability through site-specific phosphorylation
Source: Oncogene. 2023 Apr 11;42(17):1405–16. doi: 10.1038/s41388-023-02685-8 (PMC10121470; doi:10.1038/s41388-023-02685-8)
Supplement: Supplementary file 3 — Supplementary Figure Legends [file 41388_2023_2685_MOESM3_ESM.docx]

**SUPPLEMENTARY FIGURE LEGENDS**

**Supplementary Figure S1. Generation of p53S367p-specific antibody**

(A) The sequences of p53 unmodified (U) and S367-phosporylated (P) peptides.

(B) Antibody specificity was tested by dot blot analysis using increasing concentration of p53 unmodified (U) and S367p (P) peptides. Anti-p53S367p antibody was pre-incubated with p53 unmodified (U) or S367p (P) peptides in this peptide competition assay.

**Supplementary Figure S2. Altering p53 protein level and transcriptional activity after** **doxorubicin treatment**

(A) Control, VprBP-depleted, and VprBP/VprBPK194R-rescued U2OS cells were treated with doxorubicin (500 nM) for 24 h in the presence or absence of B32B3 (0.5 µM). Whole cell lysates were then prepared and analyzed by Western blotting with antibodies against p53, p53S367p and VprBP.

(B and C) Control, VprBP-depleted, and VprBP/VprBPK194R-rescued U2OS cells were treated as in (A), and subjected to RT-qPCR (B) and ChIP (C) analyses using primers listed in Supplementary Tables 1 and 2. Data represent the means ± SD of three independent experiments. *P* values were calculated using two-way ANOVA with post-hoc Tukey’s test for multiple comparisons. **P* < 0.05, ***P* < 0.01 and ****P* < 0.001 versus Ctrl sh; #*P* < 0.05, ###*P* < 0.01 and ###*P* < 0.001 versus Dox.

**Supplementary Figure S3. In vitro transcription assays with DNA templates and recombinant proteins**

p53ML DNA templates were transcribed with p53 wild-type or mutant in the presence or absence of VprBP + ATP as indicated. The radiolabeled transcripts were resolved on a 5% urea-PAGE and detected by autoradiography, and the results shown are representative of three independent experiments.

**Supplementary Figure S4.** **Blocking p53 occupancy and function at target genes by VprBP in U2OS cells**

(A) RT-qPCR was carried out as in Fig. 2C, but using primer sets for Reprimo, and PUMA genes. Data represent the means ± SD of three independent experiments. *P* values were calculated using two-way ANOVA with post-hoc Tukey’s test for multiple comparisons. ****P* < 0.001 versus Ctrl sh; ###*P* < 0.001 versus Eto.

(B) ChIP assays were as described in Fig. 2D, but using primers designed targeting Reprimo and PUMA loci. Data represent the means ± SD of three independent experiments. *P* values were calculated using two-way ANOVA with post-hoc Tukey’s test for multiple comparisons. ****P* < 0.001 versus Ctrl sh; ###*P* < 0.001 versus Eto.

**Supplementary Figure S5. p53 mRNA levels in in VprBP-depleted U2OS cells**

Total RNA was isolated from control and VprBP-depleted U2OS cells, and analyzed by RT-qPCR with p53 specific primer listed in Supplementary Table S1. Data represent the means ± SD of three independent experiments.

**Supplementary Figure S6. S367p-induced destabilization and inactivation of p53 in T84 cells**

(A) T84 cells were transfected with VprBP and/or p53 expression plasmids for 48 h in the presence or absence of B32B3 (3 µM) as indicated on the top. Extracts were prepared and analyzed by Western blotting with p53, p53S367p, FLAG, and VprBP antibodies.

(B) Total RNA was isolated from T84 cells expressing VprBP and/or p53, and subjected to RT-qPCR analysis with p21, BTG2, Reprimo and PUMA specific primers listed in Supplementary Table S1. Data represent the means ± SD of three independent experiments. *P* values were calculated using two-way ANOVA with post-hoc Tukey’s test for multiple comparisons. ****P* < 0.001 versus p53; ##*P* < 0.01 and ###*P* < 0.001 versus p53 + FLAG-VprBP.

(C) ChIP assays were performed in T84 cells expressing VprBP and/or p53 with p53 DO-1 antibody. Precipitated DNA was amplified by qPCR using primers specific for p53 response element regions in p21, BTG2, Reprimo and PUMA genes and listed in Supplementary Table S2. Data are represented as mean ± SD of three independent experiments. *P* values were calculated using two-way ANOVA with post-hoc Tukey’s test for multiple comparisons. ****P* < 0.001 versus p53; ###*P* < 0.001 versus p53 + FLAG-VprBP.

**Supplementary Figure S7. Regulatory effects of p53S367p on p53 stability and degradation**

(A) H1299 cells expressing FLAG-VprBP and p53 wild-type or S367A mutant were treated with CHX (50 µg/ml) for 0, 0.5, 1, and 2 h. Changes in p53 proteins levels were then determined by Western blot analysis of whole cell lysates. p53 band intensities were quantified using ImageJ software (ver. 1.53). Data are represented as mean ± SD of three independent experiments. *P* values were calculated using one-way ANOVA with post-hoc Bonferroni’s test for multiple comparisons. ****P* < 0.001 versus p53.

(B) H1299 cells expressing p53/VprBP were treated with MG132 (10 µM) for 6 h, and whole cell lysates were prepared and analyzed by Western blotting with anti-p53 and FLAG antibodies.

**Supplementary Figure S8. Blocking p53 occupancy and function at target genes by VprBP in H1299 cells**

(A) RT-qPCR was performed as in Fig. 3C, but using primer sets for Reprimo and PUMA genes. Data are represented as mean ± SD of three independent experiments. *P* values were calculated using two-way ANOVA with post-hoc Tukey’s test for multiple comparisons. ****P* < 0.001 versus p53; ###*P* < 0.001 versus p53 + FLAG-VprBP.

(B) ChIP assays were essentially identical to Fig. 3D, but using primers specific for Reprimo and PUMA loci. Data are represented as mean ± SD of three independent experiments. *P* values were calculated using two-way ANOVA with post-hoc Tukey’s test for multiple comparisons. ****P* < 0.001 versus p53; ###*P* < 0.001 versus p53 + FLAG-VprBP.

**Supplementary Figure S9. VprBP-driven p53 destabilization affected by S367D and 6KR mutations**

(A) H1299 cells were transfected with VprBP and p53S367D or 6KR mutant for 48 h in the presence or absence of B32B3 inhibitor. Cytoplasmic and nuclear extracts were prepared and analyzed by Western blotting with the antibodies indicated on the left.

(B) H1299 cells were transfected with p53 wild type or p53S637D or p536KR mutant and treated with MG132 (10 µM) for 6 h. Whole cell lysates were prepared and analyzed by Western blotting with anti-p53 and anti-p53ac antibodies.

**Supplementary Figure S10. Antagonistic effects of p53ac on VprBP-driven p53 inactivation**

(A) RT-qPCR was performed as in Fig. 4C, but using primer sets for Reprimo and PUMA genes. Data are represented as mean ± SD of three independent experiments. *P* values were calculated using two-way ANOVA with post-hoc Tukey’s test for multiple comparisons. ****P* < 0.001 versus p53; ###*P* < 0.001 p53 + FLAG-VprBP.

(B) ChIP assays were essentially identical to Fig. 4D, but using primers specific for Reprimo and PUMA loci. Data are represented as mean ± SD of three independent experiments. *P* values were calculated using two-way ANOVA with post-hoc Tukey’s test for multiple comparisons. ****P* < 0.001 versus p53; ###*P* < 0.001 p53 + FLAG-VprBP.

**Supplementary Figure S11. Nuclear p53 stability and activity modulated by S367p and acetylation in T84 cells**

(A) T84 cells were transfected with FLAG-VprBP and/or p53, p53S367A or p536KQ for 48 h in the presence or absence of B32B3 (3 µM). Cytoplasmic extracts and nuclear extracts were prepared and analyzed by Western blotting with indicated antibodies.

(B) Total RNA was isolated from T84 cells expressing VprBP/p53, and subjected to RT-qPCR analysis with p21, BTG2, Reprimo and PUMA specific primers. Data represent the means ± SD of three independent experiments. *P* values were calculated using two-way ANOVA with post-hoc Tukey’s test for multiple comparisons. ****P* < 0.001 versus p53; ###*P* < 0.001 p53 + FLAG-VprBP.

(C) ChIP assays were performed in T84 cells expressing VprBP/p53 with p53 DO-1 antibody and p21, BTG2, Reprimo and PUMA gene specific primers. Precipitated DNA was amplified with specific primers for p21 and BTG2 listed in Supplementary Table S2. Data are represented as mean ± SD of three independent experiments. *P* values were calculated using two-way ANOVA with post-hoc Tukey’s test for multiple comparisons. ****P* < 0.001 versus p53; ###*P* < 0.001 p53 + FLAG-VprBP.

**Supplementary Figure S12. p53 cytoplasmic translocation** **induced by VprBP-mediated S367p**

H1299 cells were co-transfected with FLAG-VprBP and/or p53 wild-type for 48 h and immunostained with p53 and VprBP antibodies as indicated on the top.

**Supplementary Figure S13.** **p53 ubiquitination and proteasomal degradation induced by VprBP-mediated S367p**

H1299 cells expressing HA-ubiquitin (Ub) were co-transfected with p53 and FLAG-VprBP wild- type or K194R mutant for 48 h and treated with MG132 (20 µM) for 6 h. Whole cell extracts were prepared, immunoprecipitated using p53 DO-1 antibody, and analyzed by Western blotting with anti-HA antibody.
