# Supplementary material for: VprBP/DCAF1 regulates p53 function and stability through site-specific phosphorylation
Source: Oncogene. 2023 Apr 11;42(17):1405–16. doi: 10.1038/s41388-023-02685-8 (PMC10121470; doi:10.1038/s41388-023-02685-8)
Supplement: Supplementary file 4 — Supplementary Experimental Procedures [file 41388_2023_2685_MOESM4_ESM.docx]

**SUPPLEMENTARY EXPERIMENTAL PROCEDURES**

**Cell lines and constructs**

U2OS and H1299 were cultured in Dulbecco’s modiﬁed Eagle’s medium and RPMI1640 medium respectively. Both media were supplemented with 10% fetal bovine serum. T84 cells were grown in DMEM/F-12 medium containing 5% fetal bovine serum. For mammalian expression of VprBP the corresponding cDNAs were amplified by PCR and ligated into the lentiviral expression vector pLenti-Hygro (Addgene, Cambridge, MA, USA) containing 5’ 3×FLAG coding sequence [1]. For recombinant VprBP expression, VprBP cDNA was ampliﬁed by PCR and inserted into the EcoRI and XhoI sites of pFASTBAC vector with an N-terminal 6×His tag to generate VprBP baculovirus expression system [2]. For transient mammalian expression of VprBP and p53, the corresponding cDNAs were amplified by PCR and ligated into the correct reading frames of pIRESneo (Clontech Laboratories, Inc., Mountain View, CA, USA) containing 5’ FLAG or untagged coding sequences [2]. For bacterial expression of p53, their cDNAs were amplified by PCR and inserted into pET11d with a 5’ FLAG tag and/or pGEX-4T1 vectors [3]. To generate mutant p53 and VprBP expression vectors, p53 and VprBP cDNAs were mutated by using Q5 Site-Directed Mutagenesis Kit (New England Biolabs, Ipswich, MA, USA) after the construction. All constructs were veriﬁed by DNA sequencing.

**Antibodies and immunostaining**

Rabbit polyclonal antibody against S367 phosphorylated p53 was developed by using the RAHSSpHLKSC peptides as an immunogen from ABclonal Inc. MA, USA. For the dot blot assay, serial dilutions of unmodified or phosphorylated peptides were spotted on a nitrocellulose membrane and blotted with the S367-phosphorylated antibody. For peptide competition assays, the antibody was pre-incubated with unmodified or phosphorylated peptides at room temperature for 60 min before immunoblotting. To access the localization of unmodified p53 and S367-phosphorylated p53, immunostaining was performed in U2OS and H1299 cells in the presence or absence of etoposide as described previously [1]. Briefly, cells were fixed with 4% (v/v) paraformaldehyde for 15 min, permeabilized with 0.1% Triton X-100 for 15 min, and immunostained with respective antibodies. Other antibodies used in this study are as follows: anti-FLAG (Sigma-Aldrich, St. Louis, MO, USA); anti-actin, anti-HA, anti-His, anti-VprBP (Proteintech, Chicago, IL, USA); anti-p53 DO-1, anti-GST (Santa Cruz Biotechnology, Dallas, TX, USA); anti-lamin (Active Motif, Carlsbad, CA, USA); anti-tubulin, anti-p53ac (Cell Signaling Technology, Beverly, MA, USA).

**Preparation of recombinant proteins**

Glutathione S-transferase (GST)-fused proteins were expressed in *E. coli* Rosetta 2 (DE3) pLysS (Novagen, Madison, WI, USA) and purified on glutathione-Sepharose 4B beads (GE Healthcare, Chicago, IL, USA) as described in our previous studies [4]. His-tagged full length VprBP was expressed in Sf21 insect cells, respectively, and puriﬁed with Nickel beads (Millipore Sigma, St. Louis, MO, USA) as detailed previously [5]. FLAG-tagged p53 was expressed in *E. coli* Rosetta 2 (DE3) pLysS cells and puriﬁed with anti-FLAG M2 agarose (Sigma-Aldrich, Sigma, St. Louis, MO, USA) according to manufacturer’s instructions. His-tagged domains of VprBP was expressed in *E. coli* Rosetta 2 (DE3) pLysS cells and puriﬁed with Ni-NTA beads (Millipore Sigma, St. Louis, MO, USA) as detailed previously [5].

***In vitro* transcription assay**

*In vitro* transcription assays were performed using p53ML601 nucleosome arrays (100 ng) for each reaction. Recombinant p53 (20 ng) and/or VprBP (50 ng) proteins were added together with ATP (10 mM). Following the transcription reactions, the radiolabeled RNAs were digested with RNase T1 and analyzed by gel electrophoresis and autoradiography.

**RNA interference**

DNA oligonucleotides encoding shRNAs speciﬁc for VprBP (CGAGAAACTGAGTCAAATGAA) coding region were annealed and ligated into the lentiviral expression vector pLKO.1 (Addgene, Cambridge, MA, USA). Lentiviral particles were generated in 293T cells by transfecting plasmids encoding VSV-G, NL-BH, and the shRNA. Two days after transfection, the soups containing viruses were collected and used to infect U2OS cells in the presence of polybrene (8 μg/ml). Cells with stable integration of shRNA constructs were selected for two weeks in the presence of puromycin (2 μg/ml). For rescue experiments, VprBP-depleted cells were infected with lentiviruses expressing shRNA-resistant VprBP, and selected for two weeks in the presence of hygromycin (500 μg/ml).

**Cell viability assay**

Cell viability was quantiﬁed using the WST-1 Cell Proliferation Reagent (Roche Diagnostics, Basel, Switzerland) according to the manufacturer’s instructions as recently described [1]. In this assay, 10 µl of WST-1 assay solution was added to each well of 96-well plate containing 100 µl of U2OS cells in the culture. After 4 h incubation at 37 °C, the relative cell viabilities were given by determining the absorbance at 460 nm at each well using a microplate reader. Each experiment was performed in triplicate.

**Protein–protein interactions**

For *in vitro* pull-down assays, GST-tagged p53 wild-type/mutant proteins (1 µg) were immobilized on Glutathione beads and incubated with His-VprBP (0.5 µg) in 500 µl of binding buffer (20 mM Tris/HCl, pH 7.3, 0.2 M KCl, 0.2 mM EDTA, 20% glycerol, 0.01% Nonidet P-40, and protease inhibitor cocktail). After washing with binding buffer, bound VprBP protein were detected by Western blotting with anti-His antibody. In another experiment, His-tagged wild- type/mutant VprBP proteins (0.5 µg) were immobilized on Ni-NTA beads and incubated with FLAG-p53 (1 µg) in 500 µl of binding buffer (20 mM HEPES-KOH, pH 7.9, 0.5 mM EDTA, 200 mM NaCl, 1 mM dithiothreitol, 10% glycerol, and 0.1% Nonidet P-40 and protease inhibitor cocktail) for 16 h at 4 °C with gentle rotation. After washing beads three times with washing buffer (20 mM HEPES-KOH, pH 7.9, 0.5 mM EDTA, 250 mM NaCl, 1 mM dithiothreitol, 10% glycerol, and 0.1% Nonidet P-40 and protease inhibitor cocktail), bound p53 proteins were detected by Western blotting with anti-FLAG antibody. For co-immunoprecipitation assays, p53 wild- type/mutant and FLAG-tagged wild-type/mutant VprBP proteins were co-expressed in H1299 cells, and whole-cell lysates were prepared from cells in cell lysis buffer (50 mM Tris/HCl,pH 7.4, 150 mM NaCl, 1 mM EDTA, 1% Triton X-100, and protease inhibitor cocktail). The cell lysates were mixed with anti-p53 DO-1 antibody conjugated to beads overnight with gentle rotation at 4°C.

**Subcellular fractionation**

H1299, U2OS and T84 cells were resuspended in cell lysis buffer (20 mM Tris-HCl, pH 7.4, 10 mM NaCl, 3mM MgCl2, 0.05% NP-40, 1 mM PMSF, and protease inhibitor cocktail). The mixture was vortexed briefly and incubated on ice for 15 min. The nuclei were pelleted by centrifugation at 1000g for 5 min at 4°C, whereas the supernatant (cytoplasmic extracts) was recovered by centrifugation at 10 000g for 20 min. Nuclei were washed with cell lysis buffer twice, resuspended in nuclear extraction buffer (10 mM Tris, pH 7.4, 100 mM NaCl, 1% Triton X-100, 1mM EDTA, 1mM EGTA, 0.1% SDS, 0.5% Sodium deoxycholate, 1 mM PMSF, 10%glycerol, and protease inhibitor cocktail), and incubated on ice for 30 min. The mixture was then centrifuged at 15 000g for 10 min, and the supernatant was collected as a nuclear extract.

**SUPPLEMENTARY REFERENCES**

1. Ghate NB, Kim S, Spiller E, Kim S, Shin Y, Rhie SK *et al*. VprBP directs epigenetic gene silencing through histone H2A phosphorylation in colon cancer. *Molecular oncology* 2021; 15: 2801-2817.

2. Kim K, Kim JM, Kim JS, Choi J, Lee YS, Neamati N *et al*. VprBP has intrinsic kinase activity targeting histone H2A and represses gene transcription. *Molecular cell* 2013; 52: 459-467.

3. Kim K, Heo K, Choi J, Jackson S, Kim H, Xiong Y *et al*. Vpr-binding protein antagonizes p53-mediated transcription via direct interaction with H3 tail. *Mol Cell Biol* 2012; 32: 783-796.

4. Kim K, Choi J, Heo K, Kim H, Levens D, Kohno K *et al*. Isolation and characterization of a novel H1.2 complex that acts as a repressor of p53-mediated transcription. *The Journal of biological chemistry* 2008; 283: 9113-9126.

5. Dyer PN, Edayathumangalam RS, White CL, Bao Y, Chakravarthy S, Muthurajan UM *et al*. Reconstitution of nucleosome core particles from recombinant histones and DNA. *Methods in enzymology* 2004; 375: 23-44.
